# An Improved Black-Winged Kite Algorithm for Global Optimization and Fault Detection

**DOI:** 10.3390/biomimetics10110728

**Published:** 2025-11-01

**Authors:** Kun Qi, Kai Wei, Rong Cheng, Guangmin Liang, Jiashun Hu, Wangyu Wu

**Affiliations:** 1 School of Electronic and Communication Engineering, Shenzhen Polytechnic University, Shenzhen 518055, China; weikai@szpu.edu.cn (K.W.); chengr@szpu.edu.cn (R.C.); jiashunhu@szpu.edu.cn (J.H.); 2 Undergraduate School of Artificial Intelligence, Shenzhen Polytechnic University, Shenzhen 518055, China; gmliang@szpu.edu.cn; 3 School of Computer Science, University of Liverpool, Liverpool L69 3DR, UK

**Keywords:** BKA, stagnation detection, adaptive guidance, fault detection

## Abstract

In the field of industrial fault detection, accurate and timely fault identification is crucial for ensuring production safety and efficiency. Effective feature selection (FS) methods can significantly enhance detection performance in this process. However, the recently proposed Black-winged Kite Algorithm (BKA) tends to suffer from premature convergence and local optima when handling high-dimensional feature spaces. To address these limitations, this paper proposes an improved Black-winged Kite Algorithm (IBKA). This algorithm integrates two novel enhancement mechanisms: First, the Stagnation-Triggered Diversification Mechanism monitors the algorithm’s convergence state and applies mild perturbations to the worst-performing individuals upon detecting stagnation, effectively preventing traps in local optima. Second, the Adaptive Weak Guidance Mechanism employs a conditional elite guidance strategy during the late optimization phase to provide subtle directional guidance to underperforming individuals, thereby improving convergence efficiency. We comprehensively evaluated the proposed IBKA across 26 benchmark functions. Results demonstrate superior performance in solution quality, convergence speed, and robustness compared to the original BKA and other advanced meta-heuristics. Furthermore, fault detection applications on public datasets validate the practical applicability of the binary version of the IBKA (bIBKA), showcasing significant improvements in detection accuracy and reliability. Experimental results confirm that these enhancement mechanisms effectively balance exploration and exploitation capabilities while preserving algorithmic simplicity and computational efficiency.

## 1. Introduction

In modern industrial production systems, equipment failures not only cause enormous economic losses but may also trigger serious safety accidents [1]. Therefore, accurate and timely fault detection has become a critical technology for ensuring industrial safety and production efficiency [2]. Industrial fault detection is essentially a complex optimization problem [3] that requires finding optimal feature data in high-dimensional feature spaces to achieve accurate identification and classification of fault patterns [4]. Meanwhile, in numerous industrial applications such as engineering design [5], parameter tuning [6], and resource allocation, the solution quality of optimization problems directly affects system performance and economic benefits. However, real-world fault detection problems often exhibit complex characteristics such as high dimensionality, multi-modality, nonlinearity, and strong coupling [7], making traditional mathematical optimization methods inadequate for handling such challenging problems.

Metaheuristic optimization algorithms [8], as a class of intelligent optimization techniques that do not rely on problem-specific mathematical properties, have demonstrated tremendous application potential in fault detection feature selection, classifier parameter optimization, and various engineering optimization problems due to their powerful global search capabilities and good adaptability [9]. These algorithms design effective search strategies to solve complex optimization problems by simulating intelligent phenomena in nature, such as biological evolution [10], swarm behavior [11], and physical processes [12]. In recent years, researchers have proposed numerous metaheuristic algorithms, including Pied Kingfisher Optimizer (PKO) [13], Red Fox Optimizer (RFO) [14], Sine Cosine Algorithm (SCA) [15], Genetic Algorithm (GA) [16], Particle Swarm Optimization (PSO) [17], Differential Evolution (DE) [18], Grey Wolf Optimizer (GWO) [19], and Whale Optimization Algorithm (WOA) [20]. Notably, Pramanik et al. [21] proposed a hybrid algorithm combining GA and GWO to select and reduce feature dimensions. Dhal et al. [22] proposed a hybrid PSO and GWO algorithm, named BGWOPSO, for binary feature selection. Abinayaa [23] proposed a hybrid optimization framework that integrates GWO with DE to address the high-dimensionality and redundancy issues in EEG signal feature selection. These algorithms have achieved significant success in industrial applications.

The Black-winged Kite Algorithm (BKA) [24] is a recently proposed bio-inspired metaheuristic optimization algorithm that achieves global optimization search by simulating the unique hovering hunting behavior and seasonal migration patterns of black-winged kites [25]. BKA abstracts the attacking behavior of black-winged kites into a local fine search mechanism and transforms their migration behavior into a global exploration strategy, theoretically possessing good exploration-exploitation balance capabilities [26]. BKA has demonstrated competitive advantages in handling standard benchmark optimization problems, laying the foundation for its application in fault detection and engineering optimization. However, in complex high-dimensional fault detection scenarios, the original BKA is prone to local optima traps [27], leading to significantly reduced detection accuracy and reliability when dealing with similar fault patterns.

To address the aforementioned problems, this paper proposes an Improved Black-winged Kite Algorithm (IBKA). The algorithm significantly enhances the optimization performance of the original BKA by integrating stagnation-triggered diversification and adaptive weak guidance mechanisms. Specifically, the main technical contributions of this research include:

1. Development of an improved BKA algorithm that integrates stagnation-triggered diversification and adaptive weak guidance mechanisms. The former employs an intelligent stagnation detection strategy to monitor the algorithm’s convergence status in real-time, systematically applying mild random perturbations to the worst-performing individuals to enhance population diversity when consecutive stagnation is detected. The latter adopts a conditionally triggered elite guidance strategy to provide subtle directional guidance from the optimal solution to poorly performing individuals in the later stages of the algorithm. The synergistic effect of these dual mechanisms effectively resolves local optima trap problems while significantly improving convergence efficiency, all while maintaining the algorithm’s exploration capabilities.
2. Comprehensive performance evaluation of IBKA and bIBKA regarding statistical significance and convergence speed. Through comparison with 9 state-of-the-art algorithms (including PSO, GWO, RFO, etc.), the superior performance of the proposed algorithms is validated.
3. Industrial fault detection application where bIBKA is applied to feature selection in fault detection, pioneering the application of bIBKA in the fault detection domain.

The organization of this paper is as follows: Section 2 presents the optimization process of the BKA algorithm. Section 3 provides detailed descriptions of the proposed IBKA and bIBKA. Section 4 presents the results and analysis of IBKA and bIBKA performance on test functions and fault detection datasets.

## 2. Black-Winged Kite Algorithm

**Figure 1:**
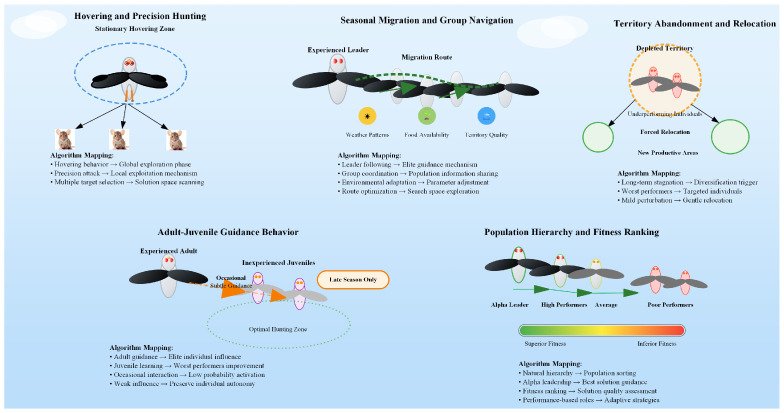
Various biological behaviors of the black-winged kite.

The BKA represents a novel nature-inspired metaheuristic optimization framework that emulates the sophisticated foraging and migratory behaviors exhibited by black-winged kites in their natural habitat [28]. The various biological behaviors of the Black-winged Kite, which form the core of the BKA, are illustrated in Figure 1. These small diurnal raptors, belonging to the family Accipitridae, demonstrate a distinctive hunting strategy characterized by sustained hovering behavior, wherein they maintain stationary flight positions at optimal altitudes while conducting aerial surveillance of the terrain below. Upon prey identification, they execute rapid vertical descents with remarkable precision to capture their quarry, demonstrating exceptional spatial awareness and timing accuracy. Beyond their hunting prowess, black-winged kites exhibit complex seasonal migration patterns that reflect their adaptive response to environmental variability and resource availability. These migratory behaviors encompass long-distance movements between breeding and wintering territories, with individuals demonstrating remarkable navigational capabilities and route optimization strategies. The migration process involves multiple decision-making stages, including departure timing, route selection, and stopover site utilization, all contributing to overall fitness and survival. The BKA algorithm incorporates these biological mechanisms through a dual-phase optimization approach: a hovering phase that simulates aerial surveillance behavior, enabling comprehensive exploration of the solution space, and a diving phase that mimics precision strike mechanisms, facilitating intensive exploitation of promising regions. Additionally, the algorithm integrates migration-inspired mechanisms for population diversity maintenance and global search capabilities, ensuring robust performance across diverse optimization landscapes. This bio-inspired approach leverages the evolutionary advantages of these avian behaviors, providing an effective balance between exploration and exploitation in complex optimization problems.

### 2.1. Population Initialization

The BKA algorithm starts by randomly generating a population of N candidate solutions and distributing them throughout the search space:

(1) $$X_{i}^{\left(0\right)}=lb+\mathrm{rand}\left(1,D\right)\times \left(ub-lb\right),\;i=1,2,\ldots ,N$$

where $X_{i}^{\left(0\right)}$ represents the initial position of the *i*-th kite, *D* is the problem dimension, lb and ub are the lower and upper bounds of the search space, respectively, and rand(1,D) generates a random vector of size *D*.

### 2.2. Attacking Behavior

The attacking behavior simulates the precision hunting strategy [29] of black-winged kites. During this phase, each kite performs local search around its current position. The mathematical formulation is:

(2) $$n\left(t\right)=0.05\times \exp\left(-2{\left(\frac{t}{T}\right)}^{2}\right)$$

where *t* is the current iteration, *T* is the maximum number of iterations, and n(t) is a time-varying parameter that controls the intensity of local search.

The position update mechanism for attacking behavior follows two strategies based on a probability parameter p=0.9:

Strategy 1 (with probability *p*):

(3) $$X_{i}^{\mathrm{new}}=X_{i}^{\left(t\right)}+n\left(t\right)\times \left(1+\sin\left(r\right)\right)\times X_{i}^{\left(t\right)}$$

Strategy 2 (with probability 1−p):

(4) $$X_{i}^{\mathrm{new}}=X_{i}^{\left(t\right)}\times \left(n\left(t\right)\times \left(2\times \mathrm{rand}\left(1,D\right)-1\right)+1\right)$$

where *r* is a random number, and rand(1,D) generates a random vector.

### 2.3. Migration Behavior

The migration behavior represents the global exploration capability of the algorithm, inspired by the seasonal movement patterns of black-winged kites. This mechanism enables the algorithm to explore distant regions of the search space and avoid local optima.

The migration factor is calculated as:

(5) $$m=2\times \sin\left(r+\frac{\pi }{2}\right)$$

The Cauchy distribution is employed to generate step sizes for migration:

(6) $$\mathrm{Cauchy}\left(x\right)=\tan\left((x-0.5)\times \pi \right)$$

where x∼U(0,1) is a uniformly distributed random variable.

The position update for migration behavior depends on the fitness comparison:

Case 1: If $f\left(X_{i}^{\left(t\right)}\right)<f\left(X_{s}^{\left(t\right)}\right)$ (current kite is better than a randomly selected kite):

(7) $$X_{i}^{\mathrm{new}}=X_{i}^{\left(t\right)}+\mathrm{Cauchy}\left(D\right)\times \left(X_{i}^{\left(t\right)}-X_{\mathrm{leader}}^{\left(t\right)}\right)$$

Case 2: If $f\left(X_{i}^{\left(t\right)}\right)\geq f\left(X_{s}^{\left(t\right)}\right)$ (current kite is worse than or equal to a randomly selected kite):

(8) $$X_{i}^{\mathrm{new}}=X_{i}^{\left(t\right)}+\mathrm{Cauchy}\left(D\right)\times \left(X_{\mathrm{leader}}^{\left(t\right)}-m\times X_{i}^{\left(t\right)}\right)$$

where $X_{s}^{\left(t\right)}$ is a randomly selected kite from the population, $X_{\mathrm{leader}}^{\left(t\right)}$ is the best solution found so far, and Cauchy(D) represents the Cauchy-distributed random vector of dimension *D*.

### 2.4. Selection Mechanism

A greedy selection mechanism is applied whenever the position is updated, either after an attacking or a migration step:

(9) $$X_{i}^{\left(t+1\right)}=\left\{\begin{array}{cc} X_{i}^{\mathrm{new}} & \mathrm{if}\;f\left(X_{i}^{\mathrm{new}}\right)<f\left(X_{i}^{\left(t\right)}\right)\\ X_{i}^{\left(t\right)} & \mathrm{otherwise} \end{array}\right.$$

where f(·) represents the objective function to be minimized.

## 3. Improved Optimization Algorithm for Black-Winged Kite Algorithm

**Figure 2:**
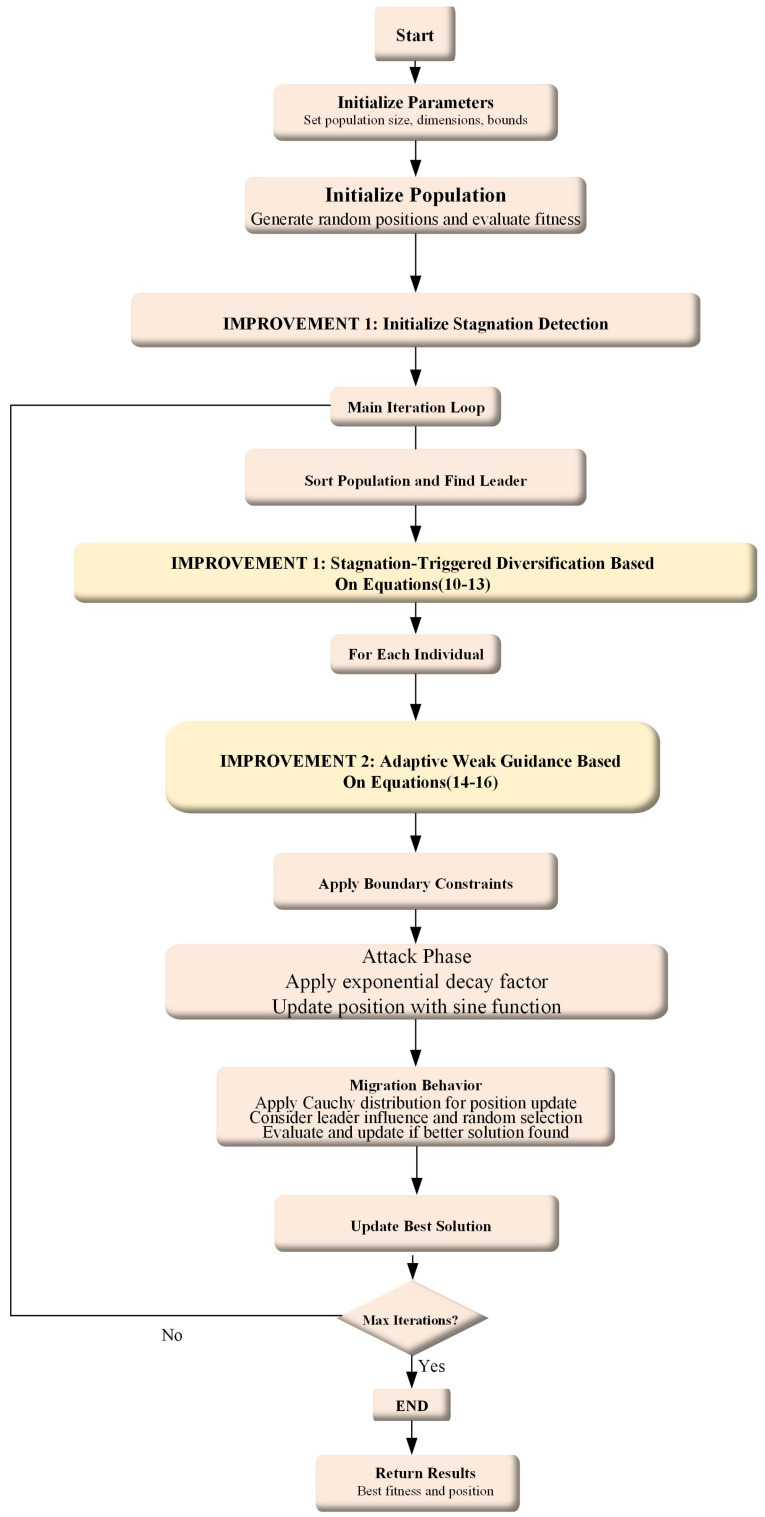
Flowchart of the IBKA.

In this section, we propose an improved Black-winged Kite Algorithm based on the original BKA to solve global optimization problems as shown in Figure 2. Building upon this, we introduce a binary version, named bIBKA, specifically designed for feature selection. Finally, bIBKA is combined with the K-Nearest Neighbor (KNN) [30] classifier and applied to the task of fault detection.

### 3.1. Enhancement Mechanisms

#### 3.1.1. Stagnation-Triggered Diversification Mechanism

The first enhancement mechanism addresses the premature convergence issue by implementing an intelligent stagnation detection and diversification strategy [31]. This mechanism monitors the algorithm’s convergence status [32] and applies targeted perturbations when stagnation is detected.

Stagnation Detection: The algorithm monitors the improvement of the best fitness value over consecutive iterations. Stagnation is identified when this improvement remains below a predefined threshold for a specified duration:

(10) $$\mathrm{Stagnation}=\left\{\begin{array}{cc} \mathrm{True} & \mathrm{if}\;|f_{\mathrm{best}}^{\left(t\right)}-f_{\mathrm{best}}^{\left(t-1\right)}|<\epsilon \;\mathrm{for}\;\tau \;\mathrm{consecutive}\;\mathrm{iterations}\\ \mathrm{False} & \mathrm{otherwise} \end{array}\right.$$

where $f_{\mathrm{best}}^{\left(t\right)}$ is the best fitness at iteration *t*, $\epsilon =1\times 10^{-15}$ is the convergence threshold, and τ=30 is the stagnation threshold.

Diversification Strategy: When stagnation is detected and the algorithm has progressed beyond the initial exploration phase (t>T/3), the mechanism selectively perturbs the worst-performing individuals to enhance population diversity:

(11) $$N_{\mathrm{div}}=\max\left(1,\left\lfloor 0.1\times N\right\rfloor \right)$$

(12) $$P_{j}=\alpha \times \left(\mathrm{ub}-\mathrm{lb}\right)\times \left(2r-1\right)$$

(13) $$X_{j}^{\mathrm{new}}=X_{j}^{\left(t\right)}+P_{j},\;j\in W$$

where $N_{\mathrm{div}}$ is the number of individuals to be diversified, α=0.02 is the perturbation strength factor, r is a random vector, and W represents the set of worst-performing individuals.

#### 3.1.2. Adaptive Weak Guidance Mechanism

The second enhancement mechanism improves convergence efficiency through a conditionally triggered elite guidance strategy that provides subtle directional guidance to underperforming individuals during the late optimization phase.

Activation Conditions: The adaptive weak guidance mechanism is activated only when all of the following conditions are met:

(14) $$\mathrm{Guidance}\;\mathrm{Active}=\left\{\begin{array}{cc} \mathrm{True} & \mathrm{if}\;i>0.85N\;\mathrm{and}\;R<0.03\;\mathrm{and}\;t>0.6T\\ \mathrm{False} & \mathrm{otherwise} \end{array}\right.$$

where *i* is the individual index (sorted by fitness), R∼U(0,1) is a random probability, and the conditions ensure that guidance is applied only to the worst 15% of individuals with a 3% probability during the final 40% of iterations.

Weak Guidance Update: When the guidance mechanism is activated, the position update incorporates a weak directional bias toward the best solution:

(15) $$D_{\mathrm{weak}}=X_{\mathrm{leader}}^{\left(t\right)}-X_{i}^{\left(t\right)}$$

(16) $$X_{i}^{\mathrm{new}}=X_{i}^{\left(t\right)}+n\left(t\right)\times \beta \times D_{\mathrm{weak}}$$

where $D_{\mathrm{weak}}$ is the direction vector toward the leader, and β=0.1 is the weak guidance strength factor that ensures the guidance remains subtle and non-disruptive.

A critical design challenge in integrating two enhancement mechanisms is ensuring they do not interfere with each other. Specifically, the potential risk exists that the weak guidance mechanism could prematurely override or undermine the diversification mechanism’s efforts. This conflict is theoretically eliminated through a multi-dimensional complementary design strategy. The mechanisms operate in distinct temporal phases to minimize interference. The Stagnation-Triggered Diversification Mechanism activates during mid-to-late optimization stages (t>T/3) upon detecting convergence stagnation, with immediate counter reset after intervention. In contrast, the Adaptive Weak Guidance Mechanism engages exclusively during the final refinement phase (t>0.6T) with ultra-low activation probability (3%). This temporal separation ensures that diversification interventions occur before guidance refinement, with negligible overlap between activation windows. The mechanisms target different subsets of the population to reduce simultaneous intervention risk. Diversification focuses on the worst-performing 10% of individuals, while weak guidance applies to the bottom 15% (i>0.85N) with stringent probabilistic control. While there exists theoretical overlap between these target ranges, the ultra-low probability of guidance activation ensures minimal simultaneous intervention. The mechanisms employ carefully calibrated intensity levels to prevent interference. Diversification uses mild perturbation strength to escape local optima without disrupting global search patterns. Weak guidance utilizes an even weaker directional bias, ensuring that even in rare concurrent activation scenarios, guidance cannot override or neutralize diversification effects. The substantial difference in intensity levels provides a safety margin against interference. The combination of temporal separation, spatial differentiation, and intensity calibration creates a hierarchical safeguard system that theoretically eliminates the risk of mechanism conflict.

### 3.2. Detailed Procedure of the IBKA

The complete IBKA algorithm integrates both enhancement mechanisms into the original BKA framework. Algorithm 1 presents the detailed pseudocode.
**Algorithm 1** The pseudocode of the IBKA**Require:** Population size *N*, Maximum iterations *T*, Problem dimension *D*, Bounds [lb,ub]
**Ensure:** Best solution $X_{\mathrm{best}}$ and fitness $f_{\mathrm{best}}$
  1:Initialize population $X_{i}$ randomly using Equation (1)  2:Evaluate fitness of all individuals  3:Initialize stagnation counter: stag_counter=0  4:Initialize last best fitness: $f_{\mathrm{last}}=\infty$  5:Set stagnation threshold: τ=30  6:t=1  7:**while** t≤T **do**  8:    Sort population by fitness and identify leader $X_{\mathrm{leader}}$  9:    Update stagnation detection using Equation (10)10:    **for** each individual i=1 to *N* **do**11:          Calculate n(t) using Equation (2)12:          **//** Attacking Behavior with Adaptive Weak Guidance13:          **if** p<r **then**14:               Apply Strategy 1 using Equation (3)15:          **else**16:               **if** Guidance conditions met (Equation (14)) **then**17:                    Apply weak guidance using Equations (15) and (16)18:               **else**19:                    Apply Strategy 2 using Equation (4)20:               **end if**21:          **end if**22:          Apply boundary handling and selection mechanism23:          **//** Migration Behavior (unchanged)24:          Apply migration behavior using Equations (7) or (8)25:          Apply boundary handling and selection mechanism26:    **end for**27:    **//** Stagnation-Triggered Diversification28:    **if** stag_counter>τ **and** t>T/3 **then**29:          Calculate $N_{\mathrm{div}}$ using Equation (11)30:          Identify worst individuals W31:          **for** each j∈W **do**32:                Generate perturbation using Equation (12)33:                Update position using Equation (13)34:                Apply boundary handling and re-evaluate fitness35:          **end for**36:          Reset stagnation counter: stag_counter=037:    **end if**38:    Update best solution if improved39:    t=t+140:**end while**41:**return** $X_{\mathrm{best}}$ and $f_{\mathrm{best}}$


### 3.3. bIBKA for Feature Selection

Given the inherent binary nature of feature selection problems, a discrete variant of the IBKA, termed bIBKA, was developed. This adaptation utilizes a transfer function to map the algorithm’s continuous search solutions into a discrete, binary feature space.

#### 3.3.1. Binary Population Initialization

Unlike continuous optimization, bIBKA initializes the population directly in binary space:

(17) $$X_{i,d}^{\left(0\right)}=\left\{\begin{array}{cc} 1 & \mathrm{if}\;R_{i,d}>0.5\\ 0 & \mathrm{otherwise} \end{array}\right.$$

where $R_{i,d}∼U\left(0,1\right)$ is a random number for the *d*-th dimension of the *i*-th individual.

#### 3.3.2. Multi-Modal Transfer Functions

bIBKA employs three different transfer functions depending on the operation context:

S-shaped Transfer Function:

(18) $$T_{S}\left(x\right)=\frac{1}{1+\exp(-2x)}$$

V-shaped Transfer Function:

(19) $$T_{V}\left(x\right)=\left|\mathrm{erf}\left(\sqrt{\frac{\pi }{2}}x\right)\right|$$

U-shaped Transfer Function:

(20) $$T_{U}\left(x\right)=\left|\sin\left(2x\right)\right|$$

#### 3.3.3. Binary Position Update Mechanisms

The binary position update varies according to the behavioral phase:

Enhanced Attacking Behavior: For attacking behavior with probability *p*:

(21) $$x_{\mathrm{cont}}=X_{i,d}^{\left(t\right)}+n\left(t\right)\times \left(1+\sin\left(r\right)\right)\times X_{i,d}^{\left(t\right)}$$

(22) $$X_{i,d}^{\mathrm{new}}=\left\{\begin{array}{cc} 1 & \mathrm{if}\;R<T_{V}\left(x_{\mathrm{cont}}\right)\\ 0 & \mathrm{otherwise} \end{array}\right.$$

Adaptive Weak Guidance (Binary Version): When guidance conditions are met:

(23) $$d_{\mathrm{weak}}=X_{\mathrm{leader},d}^{\left(t\right)}-X_{i,d}^{\left(t\right)}$$

(24) $$x_{\mathrm{cont}}=X_{i,d}^{\left(t\right)}+0.1\times n\left(t\right)\times d_{\mathrm{weak}}$$

(25) $$X_{i,d}^{\mathrm{new}}=\left\{\begin{array}{cc} 1 & \mathrm{if}\;R<T_{V}\left(x_{\mathrm{cont}}\right)\\ 0 & \mathrm{otherwise} \end{array}\right.$$

Standard Binary Conversion: For other operations:

(26) $$x_{\mathrm{cont}}=X_{i,d}^{\left(t\right)}\times \left(n\left(t\right)\times \left(2R-1\right)+1\right)$$

(27) $$X_{i,d}^{\mathrm{new}}=\left\{\begin{array}{cc} 1 & \mathrm{if}\;R<T_{S}\left(x_{\mathrm{cont}}\right)\\ 0 & \mathrm{otherwise} \end{array}\right.$$

#### 3.3.4. Binary Migration Behavior

The migration behavior is adapted for binary space using Cauchy distribution:

(28) $${\mathrm{Cauchy}}_{\mathrm{binary}}=\tan\left(\left(R-0.5\right)\times \pi \right)\times 0.1$$

(29) $$x_{\mathrm{migration}}=X_{s,d}^{\left(t\right)}+{\mathrm{Cauchy}}_{\mathrm{binary}}$$

(30) $$X_{i,d}^{\mathrm{new}}=\left\{\begin{array}{cc} 1 & \mathrm{if}\;R<T_{V}\left(x_{\mathrm{migration}}\right)\\ 0 & \mathrm{otherwise} \end{array}\right.$$

#### 3.3.5. Binary Stagnation-Triggered Diversification

The diversification mechanism in bIBKA uses bit-flip operations:

(31) $$X_{j,d}^{\mathrm{new}}=\left\{\begin{array}{cc} 1-X_{j,d}^{\left(t\right)} & \mathrm{if}\;R<\rho_{\mathrm{flip}}\\ X_{j,d}^{\left(t\right)} & \mathrm{otherwise} \end{array}\right.$$

where $\rho_{\mathrm{flip}}=0.02$ is the bit-flip probability, and j∈W represents the worst-performing individuals.

#### 3.3.6. Feature Selection Fitness Function

For feature selection problems, the fitness function combines classification accuracy and feature subset size to achieve an optimal balance between performance and complexity:

(32) $$f\left(X_{i}^{\mathrm{binary}}\right)=\alpha \times \left(1-\mathrm{Accuracy}\right)+\left(1-\alpha \right)\times \frac{|X_{i}^{\mathrm{binary}}|}{D}$$

where α∈[0,1] is a weighting parameter that balances classification accuracy and feature reduction, Accuracy is the classification accuracy obtained using the selected feature subset, $|X_{i}^{\mathrm{binary}}|$ represents the number of selected features (Hamming weight), and *D* is the total number of available features.

The fitness function is minimized, so (1−Accuracy) represents the classification error rate. The second term $\frac{|X_{i}^{\mathrm{binary}}|}{D}$ represents the ratio of selected features to total features, encouraging feature reduction. In this work, α=0.99 is used for all experiments, prioritizing classification accuracy since missing fault instances can result in severe safety consequences, while the small weight (1−α=0.01) on feature reduction encourages compact subsets when solutions achieve comparable accuracy.

### 3.4. Computational Complexity Analysis

The computational complexity of IBKA remains O(T×N×D), identical to the original BKA. The additional operations introduced by the enhancement mechanisms have negligible computational overhead:

- Stagnation detection: O(1) per iteration
- Diversification: O(0.1N×D) when triggered
- Adaptive weak guidance: O(D) for eligible individuals

## 4. Algorithm Performance Test and Analysis

### 4.1. Evaluation Criteria

The effectiveness of the proposed method is quantified using several evaluation metrics. We report the average fitness (Avg) [33], optimal fitness (Best) [34], F1-score, accuracy [35], and the standard deviation (Std) [36] to provide a thorough analysis. The formulas for these metrics can be found in Equations (33)–(37).

(33) $$\mathrm{Avg}=\frac{1}{N}\sum_{i=1}^{N}fitness_{i}$$

(34) $$\mathrm{Best}=Max(fitness_{i})$$

(35) $$\mathrm{Std}=\sqrt{\frac{1}{M-1}\sum_{i=1}^{M}{\left(fitness_{i}-\mathrm{Avg}\right)}^{2}}$$

(36) $$\text{F1-score}=\frac{2\cdot TP}{2\cdot TP+FP+FN}$$

(37) $$\mathrm{Accuracy}=\frac{TP+TN}{TP+TN+FP+FN}$$

where *N* is the number of independent runs, *M* is the total number of runs for standard deviation calculation, $fitness_{i}$ represents the fitness value of the *i*-th run, TP, TN, FP, and FN represent true positive, true negative, false positive, and false negative, respectively.

### 4.2. Experimental Settings

All algorithms were executed on a Windows 11 platform equipped with an Intel Core i5-12600K processor (3.7 GHz) and 32 GB of RAM. In Experiment I, all algorithms are configured with a population size of 30 and executed for 500 iterations in a 30-dimensional search space. For Experiment II, the population size is reduced to 10 and the number of iterations to 100. To ensure the reliability of the results, each algorithm is independently run 20 times per dataset to account for stochastic variation.

### 4.3. Experiment I: Global Optimization of IBKA Using 26 Benchmark Mathematical Functions

#### 4.3.1. Benchmark Functions

A set of 26 standard benchmark functions is employed to thoroughly evaluate the proposed algorithm’s performance on high-dimensional optimization problems. This suite is designed to test both exploitation and exploration capabilities across various search landscapes. The functions are divided into three categories: unimodal functions (F1–F7) to test convergence rate, multimodal functions (F8–F22) to assess global search and local optima avoidance, and complex composite functions (F23–F26) to evaluate the algorithm’s robustness on intricate landscapes. The complete details for these benchmark functions are summarized in Table 1.

#### 4.3.2. Experiment I: Result and Analysis

The comparative analysis of IBKA against competing algorithms, as detailed in Table 2, Table 3 and Table 4, demonstrates its effectiveness on the benchmark function set. The results show that IBKA consistently achieves favorable performance across three key metrics: Avg, Best, and Std. Regarding the Avg metric, which quantifies the expected optimization performance by computing the mean fitness across all independent runs, IBKA demonstrates superior convergence capability with extremely low average fitness values such as $7.692\times 10^{-94}$ on F9 and achieves zero or near-zero values on complex multimodal functions such as F16, F17, F19, and F26, indicating strong exploitation capability and effective global search. For the Best metric, which represents the algorithm’s ultimate optimization capacity, IBKA performs excellently by achieving the theoretical global optimum on functions F16–F19, F24, and F26 and reaching remarkable precision values such as $3.279\times 10^{-147}$ on F13 and $1.990\times 10^{-123}$ on F14, validating IBKA’s strong exploitation capability and confirming the effectiveness of the Adaptive Weak Guidance Mechanism. Concerning the Std metric, which quantifies solution stability, IBKA exhibits impressive performance with zero standard deviation on multimodal functions F16–F19, F24, and F26, indicating perfect consistency across all 20 runs, and extremely low variance on unimodal functions F1, F3, and F4, validating the effectiveness of the Stagnation-Triggered Diversification Mechanism and confirming that IBKA is highly reliable for practical engineering applications. Table 5 presents a quantitative ranking summary that clearly demonstrates IBKA’s superior performance across all evaluation metrics. IBKA achieves first place on 15 functions for average fitness, 17 for best fitness, and 14 for standard deviation—substantially outperforming all competing algorithms. Critically, IBKA ranks either first or second on 23 out of 26 functions (88.5%) for both average fitness and best fitness, demonstrating exceptional consistency. In stark contrast, the original BKA achieves first place on only 10, 8, and 10 functions respectively, while modern metaheuristics WOA and GWO secure first place on merely 5 and 4 functions for average fitness. Traditional algorithms show particularly poor performance, with GA achieving zero first-place rankings and DE failing to secure any top-three positions across all metrics. These ranking statistics provide compelling quantitative evidence that the proposed stagnation-triggered diversification and adaptive weak guidance mechanisms significantly enhance both solution quality and algorithmic stability, enabling IBKA to consistently outperform state-of-the-art competitors across diverse optimization challenges.

**Figure 3:**
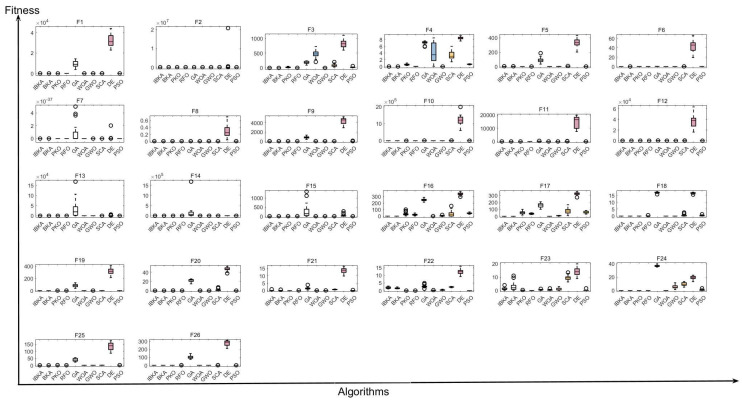
Boxplot comparison of optimization algorithms on 26 benchmark test functions.

Figure 3 presents boxplot distributions across 26 benchmark functions for performance comparison. IBKA demonstrates consistently lower median fitness values on unimodal functions F1–F4 and multimodal functions F9, F11, F20, indicating superior convergence quality. The narrow interquartile ranges on F3, F4, F16–F19, and F24–F26 confirm minimal variance across 20 runs, while significantly fewer outliers compared to GA and DE on complex functions F10–F15 validate enhanced robustness. Notably, IBKA achieves compressed box distributions on F9, F11, and F20, simultaneously demonstrating solution quality and stability. By contrast, SCA and PSO exhibit wide distributions with scattered outliers, indicating inconsistent performance. These results validate that IBKA maintains superior optimization capability and robustness across diverse problem types.

To rigorously validate the performance improvements, Wilcoxon signed-rank tests [37] were conducted for pairwise comparisons between IBKA and each competitor algorithm, as shown in Table 6. The statistical analysis reveals that IBKA consistently achieves statistically significant superiority across the vast majority of benchmark functions: PKO (25/26), RFO (25/26), GA (24/26), DE (25/26), PSO (24/26), SCA (25/26), GWO (25/26), and WOA (20/26). Remarkably, IBKA demonstrates over 90% superiority coverage for most state-of-the-art metaheuristic algorithms, with the majority exhibiting extremely strong statistical significance (*p* < 0.005). Even compared to its parent algorithm BKA, IBKA exhibits statistical significance on 15/26 functions, demonstrating substantial improvements over the baseline approach. These statistical tests provide overwhelming evidence that the observed performance gains are not attributable to random variation, but rather represent fundamental algorithmic enhancements introduced by the proposed mechanisms. The consistently high proportion of statistically significant results across diverse optimization landscapes—including unimodal, multimodal, and composite functions—confirms IBKA’s robust and generalizable superiority over existing state-of-the-art algorithms.

#### 4.3.3. Convergence Analysis

**Figure 4:**
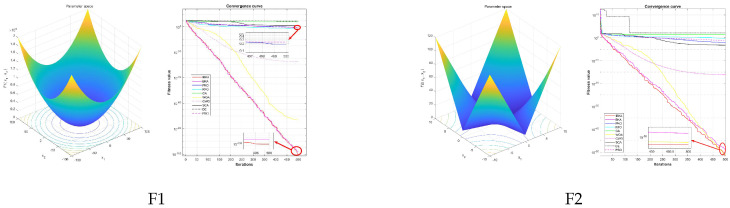

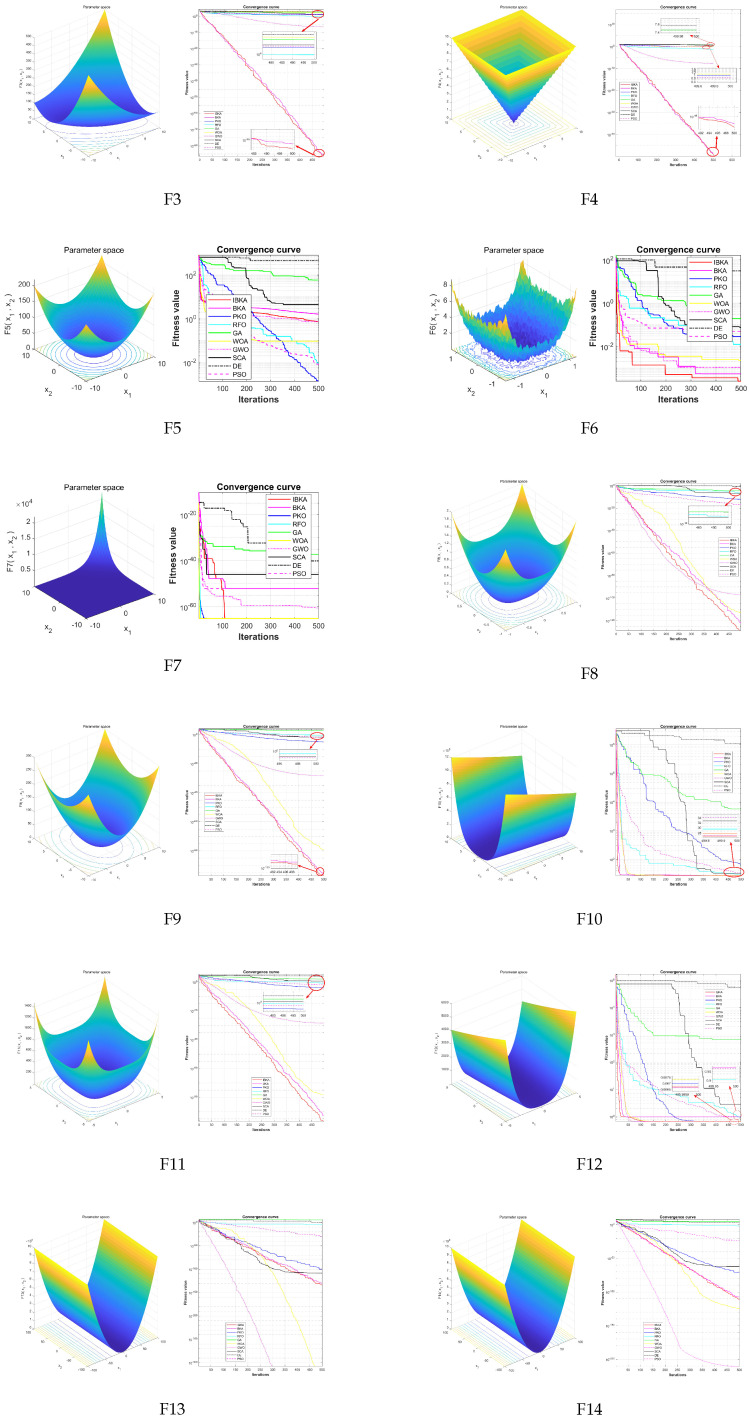

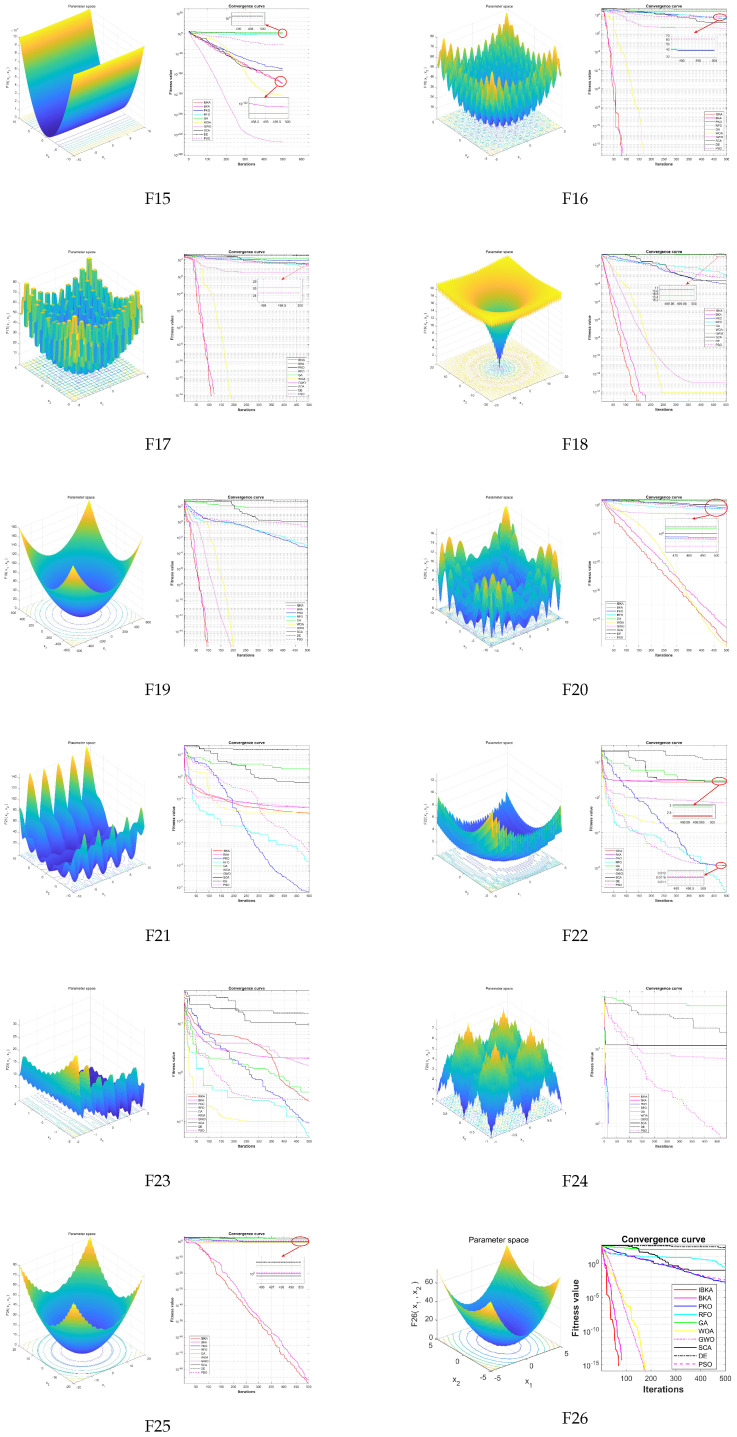
Convergence curves on each benchmark function.

To evaluate the convergence performance of the proposed algorithm, Figure 4 presents the convergence curves showing the best-so-far fitness values across iterations for all competing algorithms on 26 benchmark functions. The convergence patterns reveal distinct performance characteristics across different function types.

On unimodal functions, IBKA demonstrates rapid initial convergence followed by sustained refinement. For instance, on F1, IBKA’s curve exhibits steep descent in early iterations, reaching near-optimal regions significantly faster than competing algorithms, and maintaining stable convergence thereafter with minimal fluctuation. Similarly, on F3, IBKA’s curve is positioned substantially lower than BKA’s curve throughout the optimization process, demonstrating IBKA’s superior exploitation capability. In contrast, traditional algorithms such as GA and DE show premature stagnation on these functions, with their curves flattening at considerably higher fitness levels. On multimodal functions, IBKA exhibits robust global search capability. On F16, IBKA’s convergence curve descends sharply and reaches the bottom of the fitness scale, while competitors like PSO and SCA exhibit fluctuating curves that stabilize at visibly higher fitness levels. The smooth, monotonic descent of IBKA’s curve on F18 contrasts sharply with the irregular, oscillating patterns observed in GWO and RFO, indicating IBKA’s effective balance between exploration and exploitation. Notably, on F20, IBKA’s curve shows consistently steeper descent and lower final positioning compared to WOA, which exhibits earlier stagnation. On complex composite functions, IBKA maintains steady convergence without premature stagnation. For F25, IBKA’s curve demonstrates continuous improvement throughout all iterations, while competitors either stagnate early or converge to visibly inferior solutions. The convergence curves collectively validate that IBKA achieves faster convergence speed, superior final solution quality, and enhanced stability across diverse optimization landscapes.

### 4.4. Experiment II: Fault Detection Using bIBKA

In this experiment, we evaluated the fault detection performance of bIBK algorithm using six diverse fault datasets from different domains.The other compared algorithms are binarized as shown in the Table 7. The datasets comprise: a semiconductor manufacturing fault dataset (DS1, SECOM dataset from UCI Machine Learning Repository), a mechanical system fault dataset (DS2, from Heywhale public dataset repository), and four software fault datasets (DS3-DS6, from NASA Metrics Data Program repository) from different software projects. This heterogeneous collection of datasets enables a comprehensive assessment of bIBK’s fault detection capabilities across various industrial scenarios. Table 8 presents the detailed characteristics of these datasets.

#### 4.4.1. Experiment II: Result and Analysis

Table 9, Table 10, and Table 11 present the experimental results in terms of Avg, Accuracy, and F1-score, respectively. The results demonstrate that bIBKA exhibits superior performance compared to other baseline algorithms across most datasets, achieving notable improvements in all three metrics across semiconductor manufacturing, mechanical system, and software fault detection scenarios. Regarding the Avg metric, which quantifies the feature selection quality by combining classification error rate and feature ratio as defined in Equation (32), bIBKA performs excellently with particularly impressive results on the challenging high-dimensional semiconductor dataset DS1 and achieves consistently low values on DS2, DS3, and DS6, demonstrating superior capability in identifying compact yet informative feature subsets. For the Accuracy metric, which measures the proportion of correctly classified instances, bIBKA achieves strong classification accuracy with 72.10% on DS1 substantially surpassing bBKA, 88.90% on DS2 representing a 6.5% improvement over bBKA, 91.40% on DS3, and 86.90% on DS4, validating that bIBKA’s binary adaptation mechanisms effectively preserve the algorithm’s optimization capability in discrete search spaces. Concerning the F1-score, which provides a balanced evaluation of precision and recall particularly important for fault detection, bIBKA demonstrates excellent performance with substantial improvements over bBKA such as 0.8820 on DS2, 0.6610 on DS3, and 0.6510 on DS4, effectively addressing the precision-recall trade-off and reducing both false alarms and missed detections.

#### 4.4.2. Convergence Analysis

**Figure 5:**
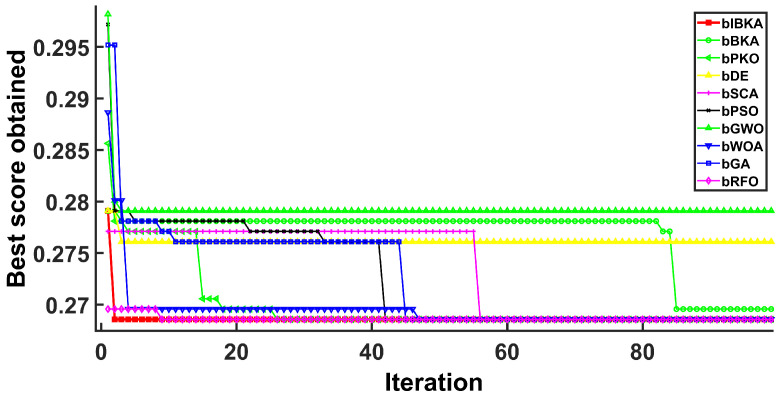
Convergence curves on DS1.

**Figure 6:**
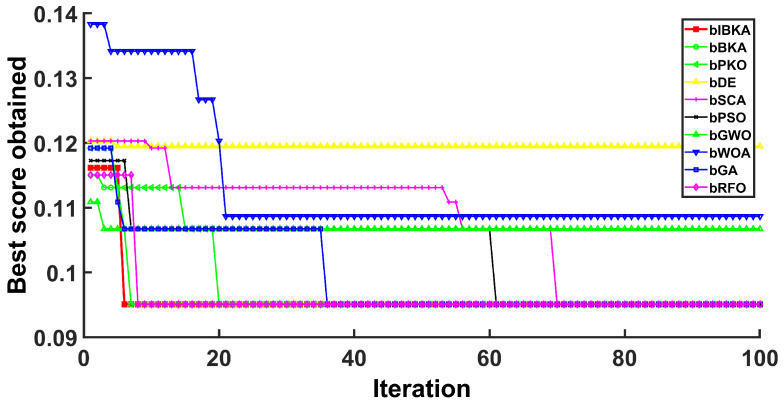
Convergence curves on DS2.

**Figure 7:**
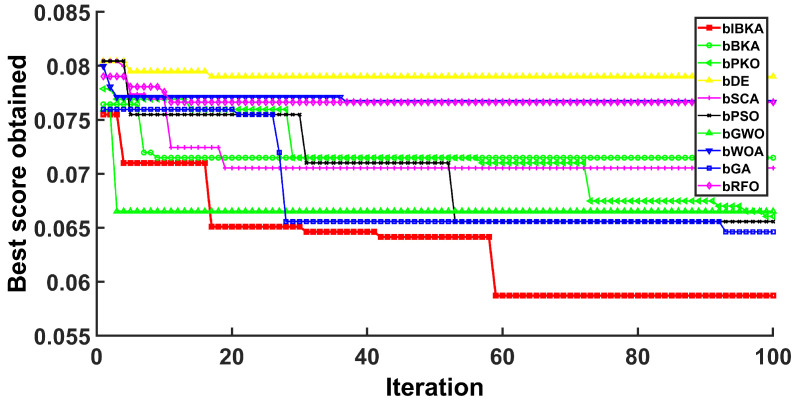
Convergence curves on DS3.

**Figure 8:**
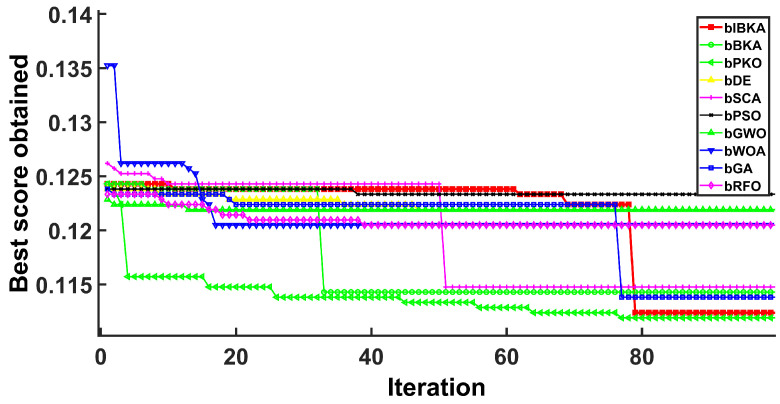
Convergence curves on DS4.

**Figure 9:**
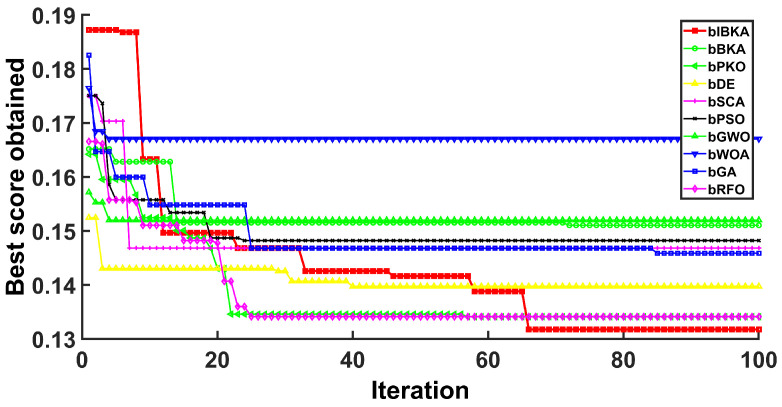
Convergence curves on DS5.

**Figure 10:**
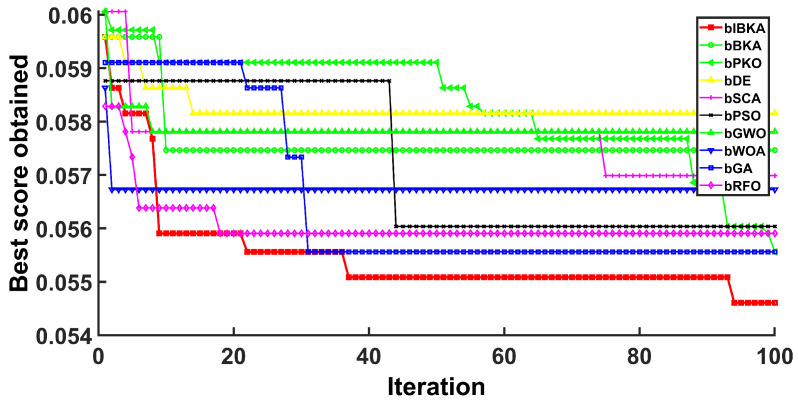
Convergence curves on DS6.

Figure 5, Figure 6, Figure 7, Figure 8, Figure 9 and Figure 10 present the convergence curves of bIBKA and competing algorithms across six fault detection datasets, tracking fitness evolution over iterations. The curves reveal dataset-specific convergence characteristics and algorithm performance differences.

On the high-dimensional semiconductor dataset DS1, bIBKA’s convergence curve exhibits rapid initial descent in early iterations, followed by gradual refinement toward the end of optimization. In comparison, bBKA’s curve is positioned noticeably higher throughout the process, while bPKO shows similar initial descent but settles at a slightly higher final level. The steeper initial slope and lower final positioning of bIBKA’s curve demonstrate superior feature selection capability in high-dimensional spaces. On DS2, bIBKA achieves the fastest convergence among all algorithms, with its curve reaching the lower fitness region earlier than competitors, whereas bSCA shows slower descent and stabilizes at a visibly higher level. The smooth, monotonic curves of bIBKA on DS3 and DS6 contrast sharply with the fluctuating, irregular patterns observed in bGA and bDE, indicating stable optimization without oscillation between local optima.

Notably, on DS4, bIBKA’s curve shows continuous improvement throughout all iterations, while bPSO and bWOA exhibit early stagnation with their curves flattening around mid-iterations. On DS5, although bPKO and bRFO’s curves are positioned slightly lower in the final region, bIBKA’s convergence curve demonstrates more consistent, steady descent with smaller iteration-to-iteration variation, suggesting better algorithmic stability. The convergence patterns across all datasets validate that bIBKA effectively balances rapid initial convergence with sustained refinement, achieving both computational efficiency and solution quality in practical fault detection scenarios.

## 5. Conclusions

This paper presents an improved Black-winged Kite Algorithm and its binary variant for addressing global optimization and fault detection challenges. The proposed Stagnation-Triggered Diversification Mechanism effectively prevents premature convergence by monitoring the population’s convergence state and applying targeted perturbations to stagnant individuals. The Adaptive Weak Guidance Mechanism further enhances optimization efficiency through a conditional elite guidance strategy, which is particularly beneficial during the late optimization phase. Comprehensive evaluations on 26 benchmark functions demonstrate IBKA’s superior performance in solution quality and convergence speed. Furthermore, experimental results across semiconductor manufacturing, mechanical system, and software fault detection scenarios validate that bIBKA achieves significant improvements in detection accuracy and reliability compared to existing algorithms while maintaining computational efficiency.

While these results validate the effectiveness of IBKA under the tested conditions, several important considerations emerge when extending this work to broader industrial contexts. The evaluation on static benchmark functions and fault datasets provides a solid foundation for understanding the algorithm’s core capabilities, though real-world deployments may encounter dynamic fault patterns where the fixed stagnation threshold could benefit from adaptive tuning mechanisms. Additionally, the fault detection datasets inherently exhibit class imbalance characteristic of real-world industrial applications, where normal operations significantly outnumber fault occurrences; in such cases, the reported accuracy values are naturally influenced by the performance of the majority class, while F1-scores provide more balanced indicators of the algorithm’s capability in detecting minority fault classes, which are often the most critical for industrial safety.

## Figures and Tables

**Table 1:** The benchmark functions.

Function Name	Formula	Range	$f_{\min}$
F1 (Sphere)	$f_{1}\left(x\right)=\sum_{i=1}^{dim}x_{i}^{2}$	[−100,100]	0
F2 (Schwefel 2.22)	$f_{2}\left(x\right)=\sum_{i=1}^{dim}|x_{i}|+\prod_{i=1}^{dim}\left|x_{i}\right|$	[−10,10]	0
F3 (Schwefel 1.2)	$f_{3}\left(x\right)=\sum_{i=1}^{dim}{\left(\sum_{j=1}^{i}x_{j}\right)}^{2}$	[−10,10]	0
F4 (Schwefel 2.21)	$f_{4}\left(x\right)=\max_{i}\left\{\right|x_{i}\left|\right\}$	[−10,10]	0
F5 (Step)	$f_{5}\left(x\right)=\sum_{i=1}^{dim}{\left|x_{i}+0.5\right|}^{2}$	[−10,10]	0
F6 (Quartic)	$f_{6}\left(x\right)=\sum_{i=1}^{dim}ix_{i}^{4}+rand$	[−1.28,1.28]	0
F7 (Exponential)	$f_{7}\left(x\right)=\exp\left(0.5\sum_{i=1}^{dim}x_{i}\right)$	[−10,10]	0
F8 (SumPower)	$f_{8}\left(x\right)=\sum_{i=1}^{dim}{\left|x_{i}\right|}^{i+1}$	[−1,1]	0
F9 (SumSquare)	$f_{9}\left(x\right)=\sum_{i=1}^{dim}ix_{i}^{2}$	[−10,10]	0
F10 (Rosenbrock)	$f_{10}\left(x\right)=\sum_{i=1}^{dim-1}\left[100{\left(x_{i+1}-x_{i}^{2}\right)}^{2}+{\left(x_{i}-1\right)}^{2}\right]$	[−10,10]	0
F11 (Zakharov)	$f_{11}\left(x\right)=\sum_{i=1}^{dim}x_{i}^{2}+\left(\frac{D}{2}\sum_{i=1}^{dim}x_{i}^{2}\right)+\left(\frac{D}{2}\sum_{i=1}^{dim}x_{i}^{4}\right)$	[−5.12,5.12]	0
F12 (Trid)	$f_{12}\left(x\right)={\left(x_{1}-1\right)}^{2}+\sum_{i=2}^{dim}i{\left(2x_{i}^{2}-x_{i-1}\right)}^{2}$	[−5,5]	0
F13 (Elliptic)	$f_{13}\left(x\right)=\sum_{i=1}^{dim}{\left(10^{6}\right)}^{\frac{i-1}{dim-1}}x_{i}^{2}$	[−100,100]	0
F14 (Cigar)	$f_{14}\left(x\right)=x_{1}^{2}+10^{6}\sum_{i=2}^{dim}x_{i}^{2}$	[−100,100]	0
F15 (Tablet)	$f_{15}\left(x\right)=10^{6}x_{1}^{2}+\sum_{i=2}^{dim}x_{i}^{6}$	[−10,10]	0
F16 (Rastrigin)	$f_{16}\left(x\right)=\sum_{i=1}^{dim}\left[x_{i}^{2}-10\cos\left(2\pi x_{i}\right)\right]+10dim$	[−5.12,5.12]	0
F17 (NCRastrigin)	$f_{17}\left(x\right)=\sum_{i=1}^{dim}y_{i}$, where $y_{i}=\left\{\begin{array}{cc} x_{i}^{2}-10\cos\left(2\pi x_{i}\right)+10 & |x_{i}|<0.5\\ {\left(\frac{round(2x_{i})}{2}\right)}^{2}-10\cos\left(2\pi \frac{round(2x_{i})}{2}\right)+10 & otherwise \end{array}\right.$	[−5.12,5.12]	0
F18 (Ackley)	$f_{18}\left(x\right)=-20\exp\left(-0.2\sqrt{\frac{1}{dim}\sum_{i=1}^{dim}x_{i}^{2}}\right)-\exp\left(\frac{1}{dim}\sum_{i=1}^{dim}\cos\left(2\pi x_{i}\right)\right)+20+e$	[−20,20]	0
F19 (Griewank)	$f_{19}\left(x\right)=\sum_{i=1}^{dim}\frac{x_{i}^{2}}{4000}-\prod_{i=1}^{dim}\cos\left(\frac{x_{i}}{\sqrt{i}}\right)+1$	[−600,600]	0
F20 (Alpine)	$f_{20}\left(x\right)=\sum_{i=1}^{dim}\left|x_{i}\sin\left(x_{i}\right)+0.1x_{i}\right|$	[−10,10]	0
F21 (Penalized1)	$f_{21}\left(x\right)=\frac{\pi }{dim}\left[10\sin^{2}\left(\pi \left(1+\frac{x_{1}+1}{4}\right)\right)+\sum_{i=1}^{dim-1}\left({\left(\frac{x_{i}+1}{4}\right)}^{2}\left(1+10\sin^{2}\left(\pi \left(1+\frac{x_{i+1}+1}{4}\right)\right)\right)\right)+{\left(\frac{x_{dim}+1}{4}\right)}^{2}\right]+\sum_{i=1}^{dim}u\left(x_{i},10,100,4\right)$	[−10,10]	0
F22 (Penalized2)	$f_{22}\left(x\right)=0.1\left[\sin^{2}\left(3\pi x_{1}\right)+\sum_{i=1}^{dim-1}{\left(x_{i}-1\right)}^{2}\left(1+\sin^{2}\left(3\pi x_{i+1}\right)\right)+{\left(x_{dim}-1\right)}^{2}\left(1+\sin^{2}\left(2\pi x_{dim}\right)\right)\right]+\sum_{i=1}^{dim}u\left(x_{i},5,100,4\right)$	[−5,5]	0
F23 (Levy)	$f_{23}\left(x\right)=\sum_{i=1}^{dim-1}{\left(x_{i}-1\right)}^{2}\left(1+\sin^{2}\left(3\pi x_{i+1}\right)\right)+\sin^{2}\left(3\pi x_{1}\right)+{\left(x_{dim}-1\right)}^{2}\left(1+\sin^{2}\left(2\pi x_{dim}\right)\right)$	[−2,2]	0
F24 (Weierstrass)	$f_{24}\left(x\right)=\sum_{i=1}^{dim}\left[\sum_{k=0}^{kmax}a^{k}\cos\left(2\pi b^{k}\left(x_{i}+0.5\right)\right)\right]-dim\sum_{k=0}^{kmax}a^{k}\cos\left(2\pi b^{k}\cdot 0.5\right)$	[−1,1]	0
F25 (Solomon)	$f_{25}\left(x\right)=1-\cos\left(2\pi \sqrt{\sum_{i=1}^{dim}x_{i}^{2}}\right)+0.1\sum_{i=1}^{dim}x_{i}^{2}$	[−20,20]	0
F26 (Bohachevsky)	$f_{26}\left(x\right)=\sum_{i=1}^{dim-1}\left[x_{i}^{2}+2x_{i+1}^{2}-0.3\cos\left(2\pi x_{i}\right)-0.4\cos\left(4\pi x_{i+1}\right)+0.7\right]$	[−5,5]	0

**Table 2:** Comparison of the average fitness of IBKA against other algorithms.

Func.	Metrics	IBKA	BKA	PKO	RFO	GA	WOA	GWO	SCA	DE	PSO
F1	Avg	$6.639\times 10^{-83}$	$1.146\times 10^{-90}$	$8.242\times 10^{-02}$	$1.052\times 10^{-02}$	$8.460\times 10^{+03}$	$3.778\times 10^{-73}$	$1.045\times 10^{-27}$	$4.182\times 10^{+00}$	$3.501\times 10^{+04}$	$3.294\times 10^{-01}$
F2	Avg	$3.417\times 10^{-38}$	$9.804\times 10^{-41}$	$1.899\times 10^{-02}$	$4.522\times 10^{-01}$	$3.125\times 10^{+01}$	$8.230\times 10^{-52}$	$1.104\times 10^{-16}$	$1.756\times 10^{-02}$	$2.608\times 10^{+05}$	$1.090\times 10^{+00}$
F3	Avg	$9.341\times 10^{-93}$	$5.482\times 10^{-73}$	$2.197\times 10^{+01}$	$7.722\times 10^{-01}$	$1.859\times 10^{+02}$	$4.534\times 10^{+02}$	$2.406\times 10^{-07}$	$8.312\times 10^{+01}$	$7.598\times 10^{+02}$	$1.786\times 10^{+01}$
F4	Avg	$1.204\times 10^{-38}$	$4.041\times 10^{-46}$	$5.659\times 10^{-01}$	$5.383\times 10^{-02}$	$7.171\times 10^{+00}$	$4.797\times 10^{+00}$	$1.327\times 10^{-07}$	$3.689\times 10^{+00}$	$8.370\times 10^{+00}$	$7.207\times 10^{-01}$
F5	Avg	$1.469\times 10^{+00}$	$1.898\times 10^{+00}$	$7.628\times 10^{-04}$	$1.060\times 10^{-02}$	$8.524\times 10^{+01}$	$8.383\times 10^{-02}$	$6.835\times 10^{-01}$	$4.996\times 10^{+00}$	$3.401\times 10^{+02}$	$2.451\times 10^{-03}$
F6	Avg	$6.068\times 10^{-04}$	$2.693\times 10^{-04}$	$4.907\times 10^{-02}$	$3.213\times 10^{-02}$	$2.899\times 10^{-01}$	$4.543\times 10^{-03}$	$1.758\times 10^{-03}$	$1.492\times 10^{-01}$	$4.150\times 10^{+01}$	$4.049\times 10^{-02}$
F7	Avg	$4.097\times 10^{-41}$	$1.988\times 10^{-32}$	$7.175\times 10^{-66}$	$7.175\times 10^{-66}$	$2.942\times 10^{-37}$	$7.175\times 10^{-66}$	$1.850\times 10^{-58}$	$1.709\times 10^{-40}$	$2.889\times 10^{-41}$	$3.834\times 10^{-54}$
F8	Avg	$6.166\times 10^{-117}$	$6.657\times 10^{-112}$	$8.385\times 10^{-10}$	$3.602\times 10^{-07}$	$3.109\times 10^{-05}$	$4.089\times 10^{-108}$	$1.060\times 10^{-97}$	$3.951\times 10^{-05}$	$2.703\times 10^{-01}$	$1.503\times 10^{-16}$
F9	Avg	$7.692\times 10^{-94}$	$1.451\times 10^{-77}$	$6.693\times 10^{-03}$	$2.183\times 10^{-01}$	$8.148\times 10^{+02}$	$1.302\times 10^{-75}$	$2.313\times 10^{-28}$	$2.602\times 10^{+00}$	$4.170\times 10^{+03}$	$5.472\times 10^{-02}$
F10	Avg	$2.793\times 10^{+01}$	$2.779\times 10^{+01}$	$3.357\times 10^{+01}$	$2.819\times 10^{+01}$	$2.923\times 10^{+03}$	$2.790\times 10^{+01}$	$2.696\times 10^{+01}$	$1.079\times 10^{+03}$	$1.207\times 10^{+06}$	$5.297\times 10^{+01}$
F11	Avg	$3.216\times 10^{-81}$	$6.104\times 10^{-79}$	$2.692\times 10^{-02}$	$1.562\times 10^{-01}$	$1.184\times 10^{+02}$	$4.962\times 10^{-75}$	$8.784\times 10^{-29}$	$8.712\times 10^{+00}$	$1.373\times 10^{+04}$	$2.037\times 10^{-02}$
F12	Avg	$6.973\times 10^{-01}$	$7.134\times 10^{-01}$	$7.568\times 10^{-01}$	$8.626\times 10^{-01}$	$9.968\times 10^{+01}$	$6.671\times 10^{-01}$	$6.667\times 10^{-01}$	$4.219\times 10^{+01}$	$4.085\times 10^{+04}$	$3.189\times 10^{+00}$
F13	Avg	$7.366\times 10^{-130}$	$6.674\times 10^{-129}$	$1.835\times 10^{-80}$	$2.845\times 10^{-05}$	$4.267\times 10^{+04}$	$0.000\times 10^{+00}$	$0.000\times 10^{+00}$	$1.380\times 10^{-94}$	$2.380\times 10^{+03}$	$1.907\times 10^{-25}$
F14	Avg	$2.567\times 10^{-108}$	$2.736\times 10^{-107}$	$1.332\times 10^{-46}$	$4.045\times 10^{-03}$	$3.243\times 10^{+04}$	$1.524\times 10^{-101}$	$9.967\times 10^{-208}$	$1.723\times 10^{-66}$	$2.248\times 10^{+03}$	$1.388\times 10^{-20}$
F15	Avg	$1.043\times 10^{-121}$	$5.494\times 10^{-123}$	$2.414\times 10^{-66}$	$1.704\times 10^{-04}$	$5.229\times 10^{+02}$	$8.836\times 10^{-130}$	$3.992\times 10^{-262}$	$1.712\times 10^{-86}$	$3.461\times 10^{+01}$	$1.650\times 10^{-27}$
F16	Avg	$0.000\times 10^{+00}$	$0.000\times 10^{+00}$	$3.055\times 10^{+01}$	$2.620\times 10^{+01}$	$2.530\times 10^{+02}$	$8.527\times 10^{-15}$	$7.319\times 10^{+00}$	$4.556\times 10^{+01}$	$3.416\times 10^{+02}$	$4.807\times 10^{+01}$
F17	Avg	$0.000\times 10^{+00}$	$0.000\times 10^{+00}$	$4.829\times 10^{+01}$	$3.323\times 10^{+01}$	$1.566\times 10^{+02}$	$0.000\times 10^{+00}$	$8.402\times 10^{+00}$	$7.327\times 10^{+01}$	$3.138\times 10^{+02}$	$4.846\times 10^{+01}$
F18	Avg	$8.882\times 10^{-16}$	$8.882\times 10^{-16}$	$6.578\times 10^{-03}$	$9.205\times 10^{-02}$	$1.675\times 10^{+01}$	$4.619\times 10^{-15}$	$9.379\times 10^{-14}$	$3.771\times 10^{-01}$	$1.651\times 10^{+01}$	$4.428\times 10^{-01}$
F19	Avg	$0.000\times 10^{+00}$	$0.000\times 10^{+00}$	$7.410\times 10^{-02}$	$1.106\times 10^{-02}$	$7.466\times 10^{+01}$	$0.000\times 10^{+00}$	$3.726\times 10^{-03}$	$1.081\times 10^{+00}$	$3.067\times 10^{+02}$	$4.346\times 10^{-01}$
F20	Avg	$2.012\times 10^{-48}$	$1.693\times 10^{-40}$	$2.028\times 10^{-02}$	$1.180\times 10^{-01}$	$2.152\times 10^{+01}$	$1.754\times 10^{-43}$	$5.267\times 10^{-04}$	$1.118\times 10^{+00}$	$4.738\times 10^{+01}$	$2.840\times 10^{-02}$
F21	Avg	$1.198\times 10^{-01}$	$6.094\times 10^{-02}$	$4.232\times 10^{-05}$	$1.766\times 10^{-04}$	$1.496\times 10^{+00}$	$1.101\times 10^{-02}$	$3.793\times 10^{-02}$	$6.608\times 10^{-01}$	$1.218\times 10^{+01}$	$5.707\times 10^{-02}$
F22	Avg	$1.820\times 10^{+00}$	$1.650\times 10^{+00}$	$4.319\times 10^{-03}$	$3.137\times 10^{-03}$	$2.944\times 10^{+00}$	$1.782\times 10^{-01}$	$3.770\times 10^{-01}$	$2.325\times 10^{+00}$	$1.298\times 10^{+01}$	$6.696\times 10^{-04}$
F23	Avg	$2.209\times 10^{+00}$	$2.513\times 10^{+00}$	$1.724\times 10^{-01}$	$3.980\times 10^{-02}$	$4.616\times 10^{-01}$	$3.270\times 10^{-01}$	$6.565\times 10^{-01}$	$9.914\times 10^{+00}$	$1.346\times 10^{+01}$	$1.679\times 10^{-01}$
F24	Avg	$0.000\times 10^{+00}$	$0.000\times 10^{+00}$	$0.000\times 10^{+00}$	$0.000\times 10^{+00}$	$3.689\times 10^{+01}$	$0.000\times 10^{+00}$	$5.607\times 10^{+00}$	$9.873\times 10^{+00}$	$1.924\times 10^{+01}$	$6.941\times 10^{-01}$
F25	Avg	$4.975\times 10^{-03}$	$4.977\times 10^{-03}$	$5.981\times 10^{-01}$	$3.570\times 10^{-01}$	$3.718\times 10^{+01}$	$1.841\times 10^{-01}$	$2.637\times 10^{-01}$	$3.732\times 10^{-01}$	$1.428\times 10^{+02}$	$9.612\times 10^{-01}$
F26	Avg	$0.000\times 10^{+00}$	$0.000\times 10^{+00}$	$1.530\times 10^{-03}$	$4.362\times 10^{-01}$	$9.334\times 10^{+01}$	$0.000\times 10^{+00}$	$0.000\times 10^{+00}$	$2.118\times 10^{-01}$	$2.713\times 10^{+02}$	$7.696\times 10^{-02}$

**Note:** Bold values indicate the best performance for the respective metric among all compared algorithms.

**Table 3:** Comparison of the best fitness of IBKA against other algorithms.

Func.	Metrics	IBKA	BKA	PKO	RFO	GA	WOA	GWO	SCA	DE	PSO
F1	Best	$1.324\times 10^{-105}$	$5.801\times 10^{-103}$	$4.791\times 10^{-05}$	$6.270\times 10^{-03}$	$3.873\times 10^{+03}$	$1.295\times 10^{-84}$	$3.367\times 10^{-29}$	$1.040\times 10^{-02}$	$2.967\times 10^{+04}$	$3.189\times 10^{-02}$
F2	Best	$8.445\times 10^{-54}$	$8.501\times 10^{-53}$	$1.249\times 10^{-04}$	$2.732\times 10^{-01}$	$2.453\times 10^{+01}$	$2.124\times 10^{-57}$	$1.853\times 10^{-17}$	$7.622\times 10^{-04}$	$8.193\times 10^{+01}$	$3.600\times 10^{-02}$
F3	Best	$3.105\times 10^{-106}$	$2.873\times 10^{-104}$	$8.909\times 10^{-01}$	$1.017\times 10^{-01}$	$8.913\times 10^{+01}$	$2.265\times 10^{+02}$	$6.433\times 10^{-11}$	$2.685\times 10^{+01}$	$4.744\times 10^{+02}$	$8.146\times 10^{+00}$
F4	Best	$5.784\times 10^{-54}$	$9.505\times 10^{-54}$	$3.157\times 10^{-01}$	$4.093\times 10^{-02}$	$6.472\times 10^{+00}$	$9.728\times 10^{-01}$	$1.512\times 10^{-08}$	$2.067\times 10^{+00}$	$7.623\times 10^{+00}$	$4.564\times 10^{-01}$
F5	Best	$5.119\times 10^{-01}$	$7.545\times 10^{-01}$	$1.027\times 10^{-05}$	$6.866\times 10^{-03}$	$3.924\times 10^{+01}$	$2.520\times 10^{-02}$	$1.056\times 10^{-04}$	$3.838\times 10^{+00}$	$2.581\times 10^{+02}$	$4.556\times 10^{-04}$
F6	Best	$6.952\times 10^{-05}$	$1.287\times 10^{-05}$	$1.169\times 10^{-02}$	$1.965\times 10^{-02}$	$1.265\times 10^{-01}$	$2.142\times 10^{-04}$	$6.834\times 10^{-04}$	$3.335\times 10^{-02}$	$2.542\times 10^{+01}$	$1.936\times 10^{-02}$
F7	Best	$7.175\times 10^{-66}$	$7.175\times 10^{-66}$	$7.175\times 10^{-66}$	$7.175\times 10^{-66}$	$2.606\times 10^{-44}$	$7.175\times 10^{-66}$	$9.680\times 10^{-66}$	$2.425\times 10^{-50}$	$6.684\times 10^{-51}$	$7.175\times 10^{-66}$
F8	Best	$1.570\times 10^{-131}$	$7.619\times 10^{-134}$	$3.173\times 10^{-20}$	$3.479\times 10^{-08}$	$2.728\times 10^{-07}$	$3.042\times 10^{-133}$	$2.338\times 10^{-104}$	$3.842\times 10^{-14}$	$7.721\times 10^{-02}$	$4.829\times 10^{-19}$
F9	Best	$5.810\times 10^{-107}$	$2.218\times 10^{-106}$	$2.808\times 10^{-06}$	$1.047\times 10^{-01}$	$2.069\times 10^{+02}$	$2.284\times 10^{-86}$	$2.734\times 10^{-30}$	$1.390\times 10^{-03}$	$3.154\times 10^{+03}$	$5.084\times 10^{-03}$
F10	Best	$2.605\times 10^{+01}$	$2.624\times 10^{+01}$	$2.712\times 10^{+01}$	$3.619\times 10^{+00}$	$1.355\times 10^{+03}$	$2.703\times 10^{+01}$	$2.539\times 10^{+01}$	$3.081\times 10^{+01}$	$7.679\times 10^{+05}$	$2.202\times 10^{+01}$
F11	Best	$4.110\times 10^{-103}$	$7.191\times 10^{-106}$	$1.354\times 10^{-05}$	$7.924\times 10^{-02}$	$2.928\times 10^{+01}$	$4.582\times 10^{-87}$	$1.173\times 10^{-30}$	$7.322\times 10^{-02}$	$7.136\times 10^{+03}$	$1.427\times 10^{-03}$
F12	Best	$6.667\times 10^{-01}$	$6.667\times 10^{-01}$	$6.667\times 10^{-01}$	$7.719\times 10^{-01}$	$2.915\times 10^{+01}$	$6.667\times 10^{-01}$	$6.667\times 10^{-01}$	$9.603\times 10^{-01}$	$2.856\times 10^{+04}$	$6.791\times 10^{-01}$
F13	Best	$3.279\times 10^{-147}$	$1.697\times 10^{-143}$	$1.162\times 10^{-136}$	$1.120\times 10^{-08}$	$2.934\times 10^{+01}$	$0.000\times 10^{+00}$	$0.000\times 10^{+00}$	$1.640\times 10^{-111}$	$2.499\times 10^{-01}$	$9.457\times 10^{-35}$
F14	Best	$1.990\times 10^{-123}$	$1.455\times 10^{-119}$	$8.096\times 10^{-75}$	$6.298\times 10^{-05}$	$2.941\times 10^{+02}$	$4.051\times 10^{-136}$	$7.981\times 10^{-254}$	$1.912\times 10^{-78}$	$2.691\times 10^{+00}$	$3.128\times 10^{-26}$
F15	Best	$6.686\times 10^{-138}$	$2.667\times 10^{-137}$	$1.019\times 10^{-103}$	$2.354\times 10^{-08}$	$8.774\times 10^{-03}$	$9.099\times 10^{-157}$	$1.729\times 10^{-291}$	$2.485\times 10^{-102}$	$1.261\times 10^{-02}$	$4.669\times 10^{-31}$
F16	Best	$0.000\times 10^{+00}$	$0.000\times 10^{+00}$	$1.359\times 10^{-01}$	$1.720\times 10^{+00}$	$2.082\times 10^{+02}$	$0.000\times 10^{+00}$	$5.684\times 10^{-14}$	$1.391\times 10^{-01}$	$2.856\times 10^{+04}$	$6.791\times 10^{-01}$
F17	Best	$0.000\times 10^{+00}$	$0.000\times 10^{+00}$	$2.051\times 10^{+00}$	$1.554\times 10^{+01}$	$1.198\times 10^{+02}$	$0.000\times 10^{+00}$	$2.309\times 10^{-14}$	$2.049\times 10^{+01}$	$2.764\times 10^{+02}$	$2.817\times 10^{+01}$
F18	Best	$8.882\times 10^{-16}$	$8.882\times 10^{-16}$	$3.918\times 10^{-04}$	$7.339\times 10^{-02}$	$1.579\times 10^{+01}$	$8.882\times 10^{-16}$	$6.839\times 10^{-14}$	$2.216\times 10^{-02}$	$1.474\times 10^{+01}$	$3.652\times 10^{-02}$
F19	Best	$0.000\times 10^{+00}$	$0.000\times 10^{+00}$	$3.807\times 10^{-04}$	$9.767\times 10^{-04}$	$3.797\times 10^{+01}$	$0.000\times 10^{+00}$	$0.000\times 10^{+00}$	$3.004\times 10^{-02}$	$2.148\times 10^{+02}$	$1.318\times 10^{-01}$
F20	Best	$9.425\times 10^{-57}$	$9.291\times 10^{-55}$	$1.799\times 10^{-03}$	$2.461\times 10^{-02}$	$1.593\times 10^{+01}$	$2.230\times 10^{-58}$	$1.107\times 10^{-17}$	$6.620\times 10^{-03}$	$3.770\times 10^{+01}$	$1.006\times 10^{-02}$
F21	Best	$1.536\times 10^{-02}$	$1.590\times 10^{-02}$	$3.645\times 10^{-07}$	$1.012\times 10^{-04}$	$4.377\times 10^{-01}$	$2.757\times 10^{-03}$	$1.221\times 10^{-02}$	$4.944\times 10^{-01}$	$8.609\times 10^{+00}$	$1.150\times 10^{-05}$
F22	Best	$7.389\times 10^{-01}$	$9.429\times 10^{-01}$	$7.311\times 10^{-06}$	$1.154\times 10^{-03}$	$1.798\times 10^{+00}$	$2.702\times 10^{-02}$	$1.962\times 10^{-04}$	$1.909\times 10^{+00}$	$7.657\times 10^{+00}$	$2.499\times 10^{-05}$
F23	Best	$2.002\times 10^{-01}$	$1.378\times 10^{-01}$	$3.320\times 10^{-02}$	$1.748\times 10^{-02}$	$2.120\times 10^{-01}$	$4.879\times 10^{-02}$	$1.153\times 10^{-01}$	$6.814\times 10^{+00}$	$1.001\times 10^{+01}$	$1.378\times 10^{-02}$
F24	Best	$0.000\times 10^{+00}$	$0.000\times 10^{+00}$	$0.000\times 10^{+00}$	$0.000\times 10^{+00}$	$3.316\times 10^{+01}$	$0.000\times 10^{+00}$	$2.452\times 10^{+00}$	$5.504\times 10^{+00}$	$1.424\times 10^{+01}$	$2.619\times 10^{-04}$
F25	Best	$1.076\times 10^{-105}$	$1.684\times 10^{-102}$	$3.980\times 10^{-01}$	$9.950\times 10^{-02}$	$2.239\times 10^{+01}$	$1.084\times 10^{-85}$	$9.950\times 10^{-02}$	$9.950\times 10^{-02}$	$9.561\times 10^{+01}$	$3.980\times 10^{-01}$
F26	Best	$0.000\times 10^{+00}$	$0.000\times 10^{+00}$	$1.018\times 10^{-06}$	$2.398\times 10^{-01}$	$6.316\times 10^{+01}$	$0.000\times 10^{+00}$	$0.000\times 10^{+00}$	$1.126\times 10^{-04}$	$1.996\times 10^{+02}$	$3.650\times 10^{-03}$

**Note:** Bold values indicate the best performance for the respective metric among all compared algorithms.

**Table 4:** Comparison of the standard deviation of IBKA against other algorithms.

Func.	Metrics	IBKA	BKA	PKO	RFO	GA	WOA	GWO	SCA	DE	PSO
F1	Std	$2.969\times 10^{-92}$	$5.008\times 10^{-90}$	$1.542\times 10^{-01}$	$3.374\times 10^{-03}$	$2.685\times 10^{+03}$	$1.588\times 10^{-72}$	$1.703\times 10^{-27}$	$8.610\times 10^{+00}$	$4.179\times 10^{+03}$	$2.171\times 10^{-01}$
F2	Std	$1.527\times 10^{-37}$	$4.385\times 10^{-40}$	$3.013\times 10^{-02}$	$9.557\times 10^{-02}$	$3.715\times 10^{+00}$	$2.020\times 10^{-51}$	$6.881\times 10^{-17}$	$2.463\times 10^{-02}$	$1.124\times 10^{+06}$	$3.067\times 10^{+00}$
F3	Std	$4.144\times 10^{-92}$	$2.452\times 10^{-72}$	$1.587\times 10^{+01}$	$7.992\times 10^{-01}$	$4.940\times 10^{+01}$	$1.110\times 10^{+02}$	$6.246\times 10^{-07}$	$4.367\times 10^{+01}$	$1.293\times 10^{+02}$	$1.057\times 10^{+01}$
F4	Std	$5.383\times 10^{-48}$	$1.807\times 10^{-45}$	$2.318\times 10^{-01}$	$9.362\times 10^{-03}$	$3.700\times 10^{-01}$	$2.817\times 10^{+00}$	$1.716\times 10^{-07}$	$9.022\times 10^{-01}$	$3.578\times 10^{-01}$	$1.284\times 10^{-01}$
F5	Std	$6.241\times 10^{-01}$	$1.074\times 10^{+00}$	$1.187\times 10^{-03}$	$2.544\times 10^{-03}$	$2.312\times 10^{+01}$	$4.421\times 10^{-02}$	$3.705\times 10^{-01}$	$1.266\times 10^{+00}$	$5.128\times 10^{+01}$	$1.838\times 10^{-03}$
F6	Std	$1.153\times 10^{-03}$	$2.397\times 10^{-04}$	$2.874\times 10^{-02}$	$1.112\times 10^{-02}$	$1.357\times 10^{-01}$	$4.015\times 10^{-03}$	$9.171\times 10^{-04}$	$1.190\times 10^{-01}$	$8.014\times 10^{+00}$	$1.171\times 10^{-02}$
F7	Std	$1.832\times 10^{-40}$	$8.889\times 10^{-32}$	$2.163\times 10^{-81}$	$2.163\times 10^{-81}$	$1.267\times 10^{-36}$	$2.163\times 10^{-81}$	$8.119\times 10^{-58}$	$7.544\times 10^{-40}$	$1.276\times 10^{-40}$	$1.715\times 10^{-53}$
F8	Std	$1.971\times 10^{-116}$	$2.935\times 10^{-111}$	$1.703\times 10^{-09}$	$3.094\times 10^{-07}$	$4.075\times 10^{-05}$	$1.498\times 10^{-107}$	$3.341\times 10^{-97}$	$7.848\times 10^{-05}$	$1.505\times 10^{-01}$	$3.289\times 10^{-16}$
F9	Std	$1.992\times 10^{-93}$	$6.490\times 10^{-77}$	$9.592\times 10^{-03}$	$6.251\times 10^{-02}$	$2.644\times 10^{+02}$	$4.516\times 10^{-75}$	$3.054\times 10^{-28}$	$4.693\times 10^{+00}$	$5.266\times 10^{+02}$	$6.672\times 10^{-02}$
F10	Std	$7.830\times 10^{-01}$	$8.081\times 10^{-01}$	$9.450\times 10^{+00}$	$5.811\times 10^{+00}$	$1.533\times 10^{+03}$	$4.236\times 10^{-01}$	$9.049\times 10^{-01}$	$1.876\times 10^{+03}$	$2.869\times 10^{+05}$	$3.359\times 10^{+01}$
F11	Std	$1.438\times 10^{-80}$	$2.730\times 10^{-78}$	$4.210\times 10^{-02}$	$5.102\times 10^{-02}$	$7.589\times 10^{+01}$	$2.166\times 10^{-74}$	$1.056\times 10^{-28}$	$1.208\times 10^{+01}$	$3.501\times 10^{+03}$	$1.719\times 10^{-02}$
F12	Std	$9.424\times 10^{-02}$	$1.141\times 10^{-01}$	$1.741\times 10^{-01}$	$7.032\times 10^{-02}$	$8.568\times 10^{+01}$	$2.922\times 10^{-04}$	$6.939\times 10^{-05}$	$1.401\times 10^{+02}$	$9.810\times 10^{+03}$	$4.967\times 10^{+00}$
F13	Std	$3.124\times 10^{-129}$	$2.118\times 10^{-128}$	$8.208\times 10^{-80}$	$4.649\times 10^{-05}$	$6.840\times 10^{+04}$	$0.000\times 10^{+00}$	$0.000\times 10^{+00}$	$5.442\times 10^{-94}$	$7.539\times 10^{+03}$	$7.322\times 10^{-25}$
F14	Std	$7.131\times 10^{-108}$	$1.208\times 10^{-106}$	$5.828\times 10^{-46}$	$5.979\times 10^{-03}$	$7.381\times 10^{+04}$	$6.816\times 10^{-101}$	$0.000\times 10^{+00}$	$5.571\times 10^{-66}$	$5.282\times 10^{+03}$	$5.749\times 10^{-20}$
F15	Std	$4.062\times 10^{-121}$	$2.450\times 10^{-122}$	$1.080\times 10^{-65}$	$2.076\times 10^{-04}$	$7.779\times 10^{+02}$	$3.951\times 10^{-129}$	$0.000\times 10^{+00}$	$7.597\times 10^{-86}$	$8.285\times 10^{+01}$	$3.863\times 10^{-27}$
F16	Std	$0.000\times 10^{+00}$	$0.000\times 10^{+00}$	$2.548\times 10^{+01}$	$1.429\times 10^{+01}$	$2.172\times 10^{+01}$	$2.082\times 10^{-14}$	$2.289\times 10^{+01}$	$5.314\times 10^{+01}$	$1.781\times 10^{+01}$	$1.509\times 10^{+01}$
F17	Std	$0.000\times 10^{+00}$	$0.000\times 10^{+00}$	$2.623\times 10^{+01}$	$1.476\times 10^{+01}$	$1.974\times 10^{+01}$	$0.000\times 10^{+00}$	$7.261\times 10^{+00}$	$2.637\times 10^{+01}$	$2.476\times 10^{+01}$	$1.360\times 10^{+01}$
F18	Std	$0.000\times 10^{+00}$	$0.000\times 10^{+00}$	$5.143\times 10^{-03}$	$1.328\times 10^{-02}$	$4.428\times 10^{-01}$	$2.438\times 10^{-15}$	$2.050\times 10^{-14}$	$6.254\times 10^{-01}$	$6.987\times 10^{-01}$	$5.254\times 10^{-01}$
F19	Std	$0.000\times 10^{+00}$	$0.000\times 10^{+00}$	$9.158\times 10^{-02}$	$8.461\times 10^{-03}$	$2.337\times 10^{+01}$	$0.000\times 10^{+00}$	$7.359\times 10^{-03}$	$6.772\times 10^{-01}$	$4.769\times 10^{+01}$	$2.341\times 10^{-01}$
F20	Std	$5.415\times 10^{-48}$	$7.573\times 10^{-40}$	$3.678\times 10^{-02}$	$2.546\times 10^{-01}$	$3.135\times 10^{+00}$	$7.844\times 10^{-43}$	$5.168\times 10^{-04}$	$1.681\times 10^{+00}$	$4.975\times 10^{+00}$	$1.686\times 10^{-02}$
F21	Std	$1.753\times 10^{-01}$	$5.684\times 10^{-02}$	$7.734\times 10^{-05}$	$4.137\times 10^{-05}$	$8.716\times 10^{-01}$	$9.238\times 10^{-03}$	$2.309\times 10^{-02}$	$1.424\times 10^{-01}$	$2.094\times 10^{+00}$	$7.870\times 10^{-02}$
F22	Std	$6.721\times 10^{-01}$	$3.934\times 10^{-01}$	$1.041\times 10^{-02}$	$2.836\times 10^{-03}$	$7.975\times 10^{-01}$	$1.126\times 10^{-01}$	$2.252\times 10^{-01}$	$1.935\times 10^{-01}$	$2.345\times 10^{+00}$	$2.442\times 10^{-03}$
F23	Std	$3.105\times 10^{+00}$	$2.746\times 10^{+00}$	$1.373\times 10^{-01}$	$1.596\times 10^{-02}$	$2.402\times 10^{-01}$	$2.997\times 10^{-01}$	$6.129\times 10^{-01}$	$1.682\times 10^{+00}$	$2.208\times 10^{+00}$	$2.234\times 10^{-01}$
F24	Std	$0.000\times 10^{+00}$	$0.000\times 10^{+00}$	$0.000\times 10^{+00}$	$0.000\times 10^{+00}$	$1.913\times 10^{+00}$	$0.000\times 10^{+00}$	$2.040\times 10^{+00}$	$1.743\times 10^{+00}$	$2.590\times 10^{+00}$	$8.867\times 10^{-01}$
F25	Std	$2.225\times 10^{-02}$	$2.225\times 10^{-02}$	$2.492\times 10^{-01}$	$1.754\times 10^{-01}$	$1.067\times 10^{+01}$	$2.148\times 10^{-01}$	$1.524\times 10^{-01}$	$2.211\times 10^{-01}$	$2.573\times 10^{+01}$	$2.992\times 10^{-01}$
F26	Std	$0.000\times 10^{+00}$	$0.000\times 10^{+00}$	$1.854\times 10^{-03}$	$2.305\times 10^{-01}$	$1.861\times 10^{+01}$	$0.000\times 10^{+00}$	$0.000\times 10^{+00}$	$4.199\times 10^{-01}$	$4.120\times 10^{+01}$	$1.475\times 10^{-01}$

**Note:** Bold values indicate the best performance for the respective metric among all compared algorithms.

**Table 5:** Performance ranking summary of algorithms across 26 benchmark functions.

Algorithm	Avg			Best			Std		
	1st	2nd	3rd	1st	2nd	3rd	1st	2nd	3rd
IBKA	**15**	**8**	2	**17**	**6**	1	**14**	**7**	3
BKA	10	7	4	8	9	5	10	6	4
PKO	3	1	2	1	0	3	3	1	2
RFO	2	3	5	1	2	4	2	3	4
GA	0	0	0	0	0	0	1	0	0
WOA	5	4	3	4	5	4	4	4	3
GWO	4	2	1	3	2	2	5	3	2
SCA	0	0	2	0	0	1	0	0	2
DE	0	0	0	0	0	0	0	0	0
PSO	1	1	4	1	1	3	1	2	3

**Note:** Numbers indicate how many functions (out of 26) each algorithm ranked 1st/2nd/3rd. For minimization problems, lower values indicate better performance.

**Table 6:** Statistical test results.

Func.	BKA	PKO	RFO	GA	WOA	GWO	SCA	DE	PSO
F1	$2.853\times 10^{-02}$	$6.796\times 10^{-08}$	$6.796\times 10^{-08}$	$6.796\times 10^{-08}$	$1.235\times 10^{-07}$	$6.796\times 10^{-08}$	$6.796\times 10^{-08}$	$6.796\times 10^{-08}$	$6.796\times 10^{-08}$
F2	$4.353\times 10^{-02}$	$6.796\times 10^{-08}$	$6.796\times 10^{-08}$	$6.796\times 10^{-08}$	$2.416\times 10^{-07}$	$6.796\times 10^{-08}$	$6.796\times 10^{-08}$	$6.796\times 10^{-08}$	$6.796\times 10^{-08}$
F3	$8.920\times 10^{-02}$	$6.796\times 10^{-08}$	$6.796\times 10^{-08}$	$6.796\times 10^{-08}$	$6.796\times 10^{-08}$	$6.796\times 10^{-08}$	$6.796\times 10^{-08}$	$6.796\times 10^{-08}$	$6.796\times 10^{-08}$
F4	$1.794\times 10^{-02}$	$6.796\times 10^{-08}$	$6.796\times 10^{-08}$	$6.796\times 10^{-08}$	$6.796\times 10^{-08}$	$6.796\times 10^{-08}$	$6.796\times 10^{-08}$	$6.796\times 10^{-08}$	$6.796\times 10^{-08}$
F5	$2.616\times 10^{-02}$	$6.796\times 10^{-08}$	$6.796\times 10^{-08}$	$6.796\times 10^{-08}$	$6.796\times 10^{-08}$	$3.293\times 10^{-05}$	$6.796\times 10^{-08}$	$6.796\times 10^{-08}$	$6.796\times 10^{-08}$
F6	$2.977\times 10^{-01}$	$6.796\times 10^{-08}$	$6.796\times 10^{-08}$	$6.796\times 10^{-08}$	$4.680\times 10^{-05}$	$6.674\times 10^{-06}$	$6.796\times 10^{-08}$	$6.796\times 10^{-08}$	$6.796\times 10^{-08}$
F7	$9.023\times 10^{-01}$	$9.029\times 10^{-08}$	$9.029\times 10^{-08}$	$4.654\times 10^{-07}$	$9.029\times 10^{-05}$	$1.993\times 10^{-02}$	$1.659\times 10^{-05}$	$7.709\times 10^{-05}$	$7.761\times 10^{-03}$
F8	$6.750\times 10^{-01}$	$6.796\times 10^{-08}$	$6.796\times 10^{-08}$	$6.796\times 10^{-08}$	$4.320\times 10^{-03}$	$6.796\times 10^{-08}$	$6.796\times 10^{-08}$	$6.796\times 10^{-08}$	$6.796\times 10^{-08}$
F9	$1.428\times 10^{-02}$	$6.796\times 10^{-08}$	$6.796\times 10^{-08}$	$6.796\times 10^{-08}$	$6.796\times 10^{-08}$	$6.796\times 10^{-08}$	$6.796\times 10^{-08}$	$6.796\times 10^{-08}$	$6.796\times 10^{-08}$
F10	$5.428\times 10^{-01}$	$3.750\times 10^{-04}$	$2.041\times 10^{-05}$	$6.796\times 10^{-08}$	$6.359\times 10^{-01}$	$2.343\times 10^{-03}$	$6.796\times 10^{-08}$	$6.796\times 10^{-08}$	$1.782\times 10^{-03}$
F11	$2.564\times 10^{-02}$	$6.796\times 10^{-08}$	$6.796\times 10^{-08}$	$6.796\times 10^{-08}$	$2.218\times 10^{-07}$	$6.796\times 10^{-08}$	$6.796\times 10^{-08}$	$6.796\times 10^{-08}$	$6.796\times 10^{-08}$
F12	$8.817\times 10^{-01}$	$1.250\times 10^{-05}$	$9.748\times 10^{-05}$	$6.796\times 10^{-08}$	$1.807\times 10^{-05}$	$4.680\times 10^{-05}$	$9.173\times 10^{-08}$	$6.796\times 10^{-08}$	$2.960\times 10^{-07}$
F13	$2.229\times 10^{-02}$	$3.416\times 10^{-07}$	$6.796\times 10^{-08}$	$6.796\times 10^{-08}$	$8.007\times 10^{-09}$	$8.007\times 10^{-09}$	$6.796\times 10^{-08}$	$6.796\times 10^{-08}$	$6.796\times 10^{-08}$
F14	$3.554\times 10^{-02}$	$6.796\times 10^{-08}$	$6.796\times 10^{-08}$	$6.796\times 10^{-08}$	$6.868\times 10^{-04}$	$6.796\times 10^{-08}$	$6.796\times 10^{-08}$	$6.796\times 10^{-08}$	$6.796\times 10^{-08}$
F15	$4.964\times 10^{-02}$	$6.796\times 10^{-08}$	$6.796\times 10^{-08}$	$6.796\times 10^{-08}$	$7.948\times 10^{-07}$	$6.796\times 10^{-08}$	$6.796\times 10^{-08}$	$6.796\times 10^{-08}$	$6.796\times 10^{-08}$
F16	$1.000\times 10^{+00}$	$8.007\times 10^{-09}$	$8.007\times 10^{-09}$	$8.007\times 10^{-09}$	$8.036\times 10^{-02}$	$7.803\times 10^{-09}$	$8.007\times 10^{-09}$	$8.007\times 10^{-09}$	$8.007\times 10^{-09}$
F17	$1.000\times 10^{+00}$	$8.007\times 10^{-09}$	$7.992\times 10^{-09}$	$8.007\times 10^{-09}$	$1.000\times 10^{+00}$	$8.007\times 10^{-09}$	$8.007\times 10^{-09}$	$8.007\times 10^{-09}$	$8.007\times 10^{-09}$
F18	$1.000\times 10^{+00}$	$8.007\times 10^{-09}$	$8.007\times 10^{-09}$	$8.007\times 10^{-09}$	$7.675\times 10^{-07}$	$7.732\times 10^{-09}$	$8.007\times 10^{-09}$	$8.007\times 10^{-09}$	$8.007\times 10^{-09}$
F19	$1.000\times 10^{+00}$	$8.007\times 10^{-09}$	$8.007\times 10^{-09}$	$8.007\times 10^{-09}$	$1.000\times 10^{+00}$	$1.980\times 10^{-02}$	$8.007\times 10^{-09}$	$8.007\times 10^{-09}$	$8.007\times 10^{-09}$
F20	$4.903\times 10^{-02}$	$6.796\times 10^{-08}$	$6.796\times 10^{-08}$	$6.796\times 10^{-08}$	$8.357\times 10^{-04}$	$6.796\times 10^{-08}$	$6.796\times 10^{-08}$	$6.796\times 10^{-08}$	$6.796\times 10^{-08}$
F21	$5.428\times 10^{-01}$	$6.796\times 10^{-08}$	$6.796\times 10^{-08}$	$1.657\times 10^{-07}$	$1.918\times 10^{-07}$	$4.320\times 10^{-03}$	$1.235\times 10^{-07}$	$6.796\times 10^{-08}$	$6.389\times 10^{-02}$
F22	$5.250\times 10^{-01}$	$6.796\times 10^{-08}$	$6.796\times 10^{-08}$	$1.190\times 10^{-04}$	$6.796\times 10^{-08}$	$7.898\times 10^{-08}$	$7.718\times 10^{-03}$	$6.796\times 10^{-08}$	$6.796\times 10^{-08}$
F23	$7.150\times 10^{-01}$	$2.960\times 10^{-07}$	$6.796\times 10^{-08}$	$1.190\times 10^{-04}$	$1.104\times 10^{-05}$	$7.718\times 10^{-03}$	$2.356\times 10^{-06}$	$7.948\times 10^{-07}$	$1.918\times 10^{-07}$
F24	$1.000\times 10^{+00}$	$1.000\times 10^{+00}$	$1.000\times 10^{+00}$	$8.007\times 10^{-09}$	$1.000\times 10^{+00}$	$8.007\times 10^{-09}$	$8.007\times 10^{-09}$	$8.007\times 10^{-09}$	$8.007\times 10^{-09}$
F25	$4.903\times 10^{-01}$	$6.796\times 10^{-08}$	$6.796\times 10^{-08}$	$6.796\times 10^{-08}$	$3.288\times 10^{-05}$	$6.796\times 10^{-08}$	$6.796\times 10^{-08}$	$6.796\times 10^{-08}$	$6.796\times 10^{-08}$
F26	$1.000\times 10^{+00}$	$8.007\times 10^{-09}$	$8.007\times 10^{-09}$	$8.007\times 10^{-09}$	$1.000\times 10^{+00}$	$1.000\times 10^{+00}$	$8.007\times 10^{-09}$	$8.007\times 10^{-09}$	$8.007\times 10^{-09}$

**Note:** Bold values indicate significant difference from IBKA.

**Table 7:** Binarization methods for comparison algorithms.

Algorithm	Binarization Method
bPSO	Sigmoid transfer function
bDE	Threshold method (θ=0.5)
bGWO	Modified sigmoid function
bWOA	Threshold method (θ=0.5)
bSCA	Threshold method (θ=0.5)
bGA	Sigmoid transfer function
bBKA	Sigmoid transfer functionn
bIBKA	Adaptive multi-transfer functions (proposed: Section 3.3.2)
bPKO	Threshold method (θ=0.5)
bRFO	Sigmoid transfer function

**Table 8:** Summary of fault detection datasets.

Dataset	Domain	Features	Instances	Class Distribution
DS1	Semiconductor	591	1567	Normal: 93.4%, Faulty: 6.6%
DS2	Mechanical	9	943	Normal: 69.14%, Faulty: 30.86%
DS3	Software	21	3301	Normal: 68.95%, Faulty: 31.05%
DS4	Software	21	3301	Normal: 69.34%, Faulty: 30.66%
DS5	Software	21	3301	Normal: 68.62%, Faulty: 31.38%
DS6	Software	21	3302	Normal: 69.05%, Faulty: 30.95%

**Table 9:** Average Avg of feature subsets obtained by bIBKA versus other competing algorithms.

Datasets	bIBKA	bBKA	bPKO	bDE	bSCA	bPSO	bGWO	bWOA	bGA	bRFO
DS1	**0.7210**	0.6370	0.7150	0.6720	0.6810	0.6728	0.6636	0.6720	0.3150	0.6720
DS2	**0.0960**	**0.0960**	0.0980	0.1000	0.1190	0.1020	0.1060	0.1130	0.0990	**0.0960**
DS3	**0.0740**	0.0810	**0.0740**	0.0850	0.0790	0.0810	0.0840	0.0830	0.0790	0.0751
DS4	**0.1120**	0.1170	0.1140	0.1190	0.1220	0.1230	0.1210	0.1210	0.1200	0.1200
DS5	0.1430	0.1520	**0.1380**	0.1480	0.1400	0.1500	0.1520	0.1670	0.1490	**0.1380**
DS6	**0.0520**	0.0570	0.0550	0.0570	0.0580	0.0570	0.0570	0.0560	0.0560	0.0560

**Note:** Bold values indicate the best performance for the respective metric among all compared algorithms.

**Table 10:** Average accuracy of feature subsets obtained by bIBKA versus other competing algorithms.

Datasets	bIBKA	bBKA	bPKO	bDE	bSCA	bPSO	bGWO	bWOA	bGA	bRFO
DS1	**0.7210**	0.6370	0.7150	0.6720	0.6810	0.6728	0.6636	0.6720	0.3150	0.6720
DS2	**0.8890**	0.8240	0.8820	0.8880	0.8770	0.8880	0.8820	0.8610	0.8880	0.8880
DS3	**0.9140**	0.9040	0.9090	0.9090	0.9090	0.9000	0.9040	0.9090	0.9090	0.9040
DS4	**0.8690**	0.8280	0.8480	0.8680	0.8280	0.8580	0.8280	0.8380	0.8580	0.8380
DS5	0.7720	0.7600	0.7710	0.7860	0.7760	0.7760	**0.7980**	0.7880	0.7760	0.7710
DS6	**0.9480**	0.9380	0.9400	0.9450	0.9400	0.9430	0.9430	0.9430	0.9430	0.9400

**Note:** Bold values indicate the best performance for the respective metric among all compared algorithms.

**Table 11:** Average F1-score of feature subsets obtained by bIBKA versus other competing algorithms.

Datasets	bIBKA	bBKA	bPKO	bDE	bSCA	bPSO	bGWO	bWOA	bGA	bRFO
DS1	0.5889	0.5700	0.5880	0.5880	0.6180	0.5880	0.5860	0.5880	**0.6300**	0.5880
DS2	**0.8820**	0.8140	0.8630	0.8720	0.8700	0.8800	0.8750	0.8530	**0.8820**	**0.8820**
DS3	**0.6610**	0.5940	0.6450	0.6160	0.6310	0.6020	0.5940	0.6590	0.6310	0.6480
DS4	**0.6510**	0.5640	0.5790	0.6430	0.5140	0.5970	0.5530	0.5640	0.6240	0.5640
DS5	0.6360	0.5880	0.6260	0.6310	0.6260	0.6220	**0.6600**	0.6330	0.6260	0.6260
DS6	**0.7010**	0.5820	0.5720	0.6770	0.5720	0.6030	0.6030	0.5910	0.6160	0.4840

**Note:** Bold values indicate the best performance for the respective metric among all compared algorithms.

## Data Availability

The data that support the findings of this study are available from the corresponding authors upon reasonable request.
